# Integrating optimization and machine learning for estimating water resistivity and saturation in shaley sand reservoirs

**DOI:** 10.1038/s41598-026-36133-w

**Published:** 2026-02-11

**Authors:** Muhammad A. El Hameedy, Walid M. Mabrouk, Ahmed M. Metwally

**Affiliations:** Department of Geophysics, Faculty of Science, Cairo university, Giza, 12613 Egypt

**Keywords:** Shaley-Sand reservoirs, Water resistivity estimation, Optimization, Machine learning, Neural networks, Ensemble learning, Engineering, Mathematics and computing, Solid Earth sciences

## Abstract

Accurate characterization of shaley-sand reservoirs remains a significant challenge in petroleum geophysics, where complex clay mineralogy often renders traditional evaluation methods unreliable. This study introduces an integrated, data-driven framework that synergizes numerical optimization and machine learning (*ML*) to accurately estimate formation water resistivity (*R*_*w*_) and predict water saturation (*S*_*w*_), overcoming the limitations of data scarcity. The workflow begins with rigorous preprocessing of well log data from 11 wells across the Norwegian North Sea and Egyptian Western Desert. First, we establish a robust, physically-constrained *R*_*w*_ by evaluating four optimization algorithms. The Powell and Nelder-Mead algorithms emerged as superior, demonstrating the ability to recover the true Rw from log data with low error (1×10^-4^ RMSE) against measured samples rapidly. This optimized *R*_*w*_ then serves as a high-quality "pseudo-core" label to generate a continuous *S*_*w*_ log for training a comprehensive suite of ML models, including ensemble methods (Random Forest, CatBoost, XGBoost) and neural networks (ANNs, LSTM), to predict *S*_*w*_. The models demonstrated predictive accuracy, validated by a robust 5-fold cross-validation protocol. On the blind test wells, the top-performing models (LSTM, CatBoost , and XGBoost) achieved a coefficient of determination (R^2^) up to 0.944 with Mean Absolute Error (*MAE*) and Root Mean Squared Error (*RMSE*) as low as 0.03 and 0.050 respectively. The automated fusion of optimization-derived physics with ML-driven prediction marks a transformative step toward more reliable, data-centric petrophysical workflows. This integrated framework offers a significant enhancement in reservoir characterization, providing a cost-effective and scalable methodology that reduces reliance on expensive core analyses and improves the accuracy of hydrocarbon-in-place estimations.

**Supplementary Information:**

The online version contains supplementary material available at 10.1038/s41598-026-36133-w.

## Introduction

Shaley sandstone reservoirs constitute a critical component of global hydrocarbon resources, contributing substantially to the world’s energy supply due to their substantial hydrocarbon-bearing potential^1,2^.However, their exploitation is complicated by inherent heterogeneity, complex pore geometries, and variable clay mineralogy, which collectively impedes accurate petrophysical evaluation. Effective reservoir characterization in such formations hinges on the accurate quantification of two key parameters: water saturation (*S*_*w*_) and formation water resistivity (*R*_*w*_). *S*_*w*_ - defined as the proportion of pore volume occupied by aqueous phases - serves as a critical petrophysical parameter for hydrocarbon volume estimation, reservoir performance forecasting, and economic feasibility assessments^3^. A persistent challenge arises from the conductive behavior of electrochemically active clay minerals (e.g., smectite, illite), whose cation exchange capacity (CEC) induces excess electrical conductivity in the pore network. This phenomenon deviates sharply from the assumptions of conventional clean-sand models such as Archie’s equation^4^, which neglects clay-related conductivity effects. Consequently, reliance on unmodified Archie-type models in shaley sandstone reservoirs often results in systematic overestimation of *S*_*w*_, leading to undervalued hydrocarbon reserves and suboptimal field development strategies^5^.

The influence of shale on *S*_*w*_ demands specialized petrophysical interpretation methodologies. Although laboratory-based direct measurements of *S*_*w*_ (core analysis) offer high precision, they are prohibitively expensive, time-intensive, and restricted to discrete depth intervals, limiting their utility for continuous reservoir evaluation^6^. Consequently, well-log-derived petrophysical analysis has emerged as a practical alternative, enabling continuous in-situ quantification of reservoir properties - including porosity, permeability, and hydrocarbon saturation - along the borehole column^7–11^. However, the accuracy of log-based *S*_*w*_ models is inherently contingent on precise determination of *R*_*w*_, as uncertainties in *R*_*w*_ propagate into *S*_*w*_ estimations, compromising reservoir assessments.

Multiple empirical models have been proposed over the past few decades, to account for clay-induced conductivity effects in *S*_*w*_ calculations for shaley reservoirs. Seminal methodologies include the DeWitte model^12^, Simandoux equation^13^, Waxman-Smits model^5^, and Indonesia model^14^, each designed to address distinct shale distribution patterns (e.g., dispersed, laminated, or structural clays). While these models improve *S*_*w*_ estimation accuracy in clay-rich formations compared to conventional clean-sand approaches, their applicability remains limited by lithological dependencies. Critically, they exhibit high sensitivity to input parameters such as *R*_*w*_ and shale volume (*V*_*sh*_), which are often subject to measurement inaccuracies. Furthermore, their reliance on idealized assumptions about clay geometry and mineralogy restricts their utility to specific reservoir types, rendering them ineffective in highly heterogeneous or mixed lithologies. These limitations, compounded by inherent uncertainties in model-specific parameters (e.g., cation exchange capacity, and clay conductivity), frequently lead to misinterpretations of *S*_*w*_, as underscored in critical reviews of their practical applicability^15^.

On the other hand, graphical methods for *R*_*w*_ determination, such as Hingle and Pickett plots^16,17^, similarly suffer from oversimplified petrophysical assumptions. The Hingle plot, for instance, presupposes shale-free lithologies and linear resistivity-porosity relationships, rendering it unreliable in complex formations such as shaly sands or carbonates. Its subjective graphical interpretation introduces additional variability in heterogeneous reservoirs, while errors in porosity or resistivity measurements propagate nonlinearly into erroneous *R*_*w*_ estimates. The Pickett plot, rooted in Archie’s equation^4^, requires calibration using water-bearing zones - a process prone to misidentification in multiphase or unconventional reservoirs^18^. Both methods fail to account for conductive minerals, organic matter, or data noise, significantly limiting their robustness in modern reservoirs with intricate mineralogy. Such shortcomings highlight the inadequacy of traditional graphical techniques in contemporary, data-driven petrophysical workflows.

Existing numerical methods for *R*_*w*_ estimation exhibit critical limitations in applicability and accuracy. For instance, Authors^19^ developed a deterministic approach for *R*_*w*_ and water saturation (*S*_*w*_) determination, achieving high precision in clean reservoirs; however, their methodology fails to account for shale effects, rendering it unsuitable for clay-rich formations. Authors^20^ extended this work to shaley reservoirs, reporting a root mean square error (RMSE) of >10 % and < 2.1% for *R*_*w*_ and *S*_*w*_ predictions, respectively. Yet, their validation relied on sparse data from the Surma Basin (Bengal), without generalizability across diverse lithologies/basins and fluid types. To overcome these constraints, this study proposes a novel framework leveraging numerical optimization techniques to calculate *R*_*w*_ solely from three well-log inputs: neutron porosity (ϕ_*N*_), gamma ray-derived shale volume (*V*_*sh*_), and deep resistivity (*R*_*t*_). The methodology optimizes *R*_*w*_ by minimizing the difference between measured *R*_*t-meas*_ and calculated *R*_*t-calc*_ via the Schlumberger shaley-sand *S*_*w*_ equation^21^, explicitly incorporating variable fluid saturations (water, oil, condensate, gas) across heterogeneous formations. This approach eliminates the dependency on core-calibrated parameters or idealized lithological assumptions, offering a robust and generalized, data-driven alternative to conventional methods especially when integrated with Machine learning (*ML*) workflows.

Regarding this manner, Emergence of ML as a transformative approach for water saturation (*S*_*w*_) prediction, overcoming the limitations of traditional empirical models, which are often constrained by region-specific assumptions, parameter uncertainties, and poor generalizability. Among ML techniques, Artificial Neural Networks (ANN) and Fuzzy Logic (FL) have gained prominence, with ANN demonstrating exceptional versatility across diverse lithologies and fluid systems. Early innovations by^22^ leveraged committee neural networks to integrate sonic, density, neutron porosity, and resistivity logs to predict *S*_*w*_, demonstrating superior accuracy compared to conventional petrophysical models. However, their methodology had a critical oversight: the exclusion of shale effects, which are essential for accurate *S*_*w*_ estimation in clastic reservoirs. This omission limits the framework applicability to heterogeneous shale-bearing formations, Authors^23^further improved predictive accuracy by incorporating self-potential (SP) logs alongside gamma ray, deep resistivity, neutron, and density measurements. Subsequent advancements focused on optimizing input selection and model architecture^24^:employed resilient backpropagation ANNs with feature ranking to prioritize critical log inputs (density, neutron, resistivity, and photoelectric factor), while^25^ utilized hybrid core-log datasets to calibrate saturation exponents (*n*) and cementation factors (*m*), demonstrating ANN superiority over conventional dual-water models.

Authors^26^ applied Levenberg-Marquardt-trained ANNs to quantify the sensitivity of log variables (e.g., GR, R_t_, ϕ_N_) to reservoir parameters, validating ANN capability to predict not only *S*_*w*_ and porosity (ϕ) but also composite petrophysical indices. Region-specific applications, such as^27^ feed-forward backpropagation ANN for *S*_*w*_, ϕ, and permeability (k) prediction in the Niger Delta, achieved high accuracy using minimal inputs (GR, density, and R_t_). Most recently, Authors^28^ derived an empirical *S*_*w*_ correlation directly from ANN weight matrices, optimizing predictions in shale-rich formations using Vsh, Rt, ϕ, and k logs. Collectively, these studies underscore ANN adaptability, accuracy, and capacity to bypass restrictive assumptions inherent to conventional models.

In Middle Eastern carbonate reservoirs, hybrid machine learning (ML) frameworks integrating ANN with FL have demonstrated significant advantages over standalone empirical and ANN models. For instance^29^, reported that the Adaptive Neuro-Fuzzy Inference System (ANFIS) achieved marginally higher accuracy than ANN in *S*_*w*_ prediction tasks, while^30^ observed FL’s superior performance over ANN in similar lithologies. Further innovations in ANN optimization have enhanced computational efficiency and robustness: Authors^31^ integrated ANN with an Imperialist Competitive Algorithm (ICA) for unconventional reservoirs, emphasizing that outlier detection and noise reduction improved prediction reliability. Similarly, study^32^ utilized radial basis function (RBF) neural networks in Iran’s Sarvak Formation, noting RBF’s rapid convergence and structural simplicity as key advantages over traditional ANN architectures. Collectively, hybrid systems such as ANFIS, ICA-ANN, and RBF-ANN have advanced reservoir modeling by balancing generalization capacity with computational efficiency.

In parallel, FL networks have emerged as competitive tools for petrophysical prediction. Authors^6^ demonstrated FL computational efficiency and deterministic solutions for *S*_*w*_ and ϕ estimation, contrasting with ANN stochastic training processes. Authors^33^ refined FL accuracy by testing metaheuristic optimizers - including Differential Evolution, Particle Swarm Optimization (PSO), and Covariance Matrix Adaptation Evolution Strategy (CMAES) - with PSO achieving the highest predictive stability. These advancements underscore FL adaptability to complex reservoir systems, particularly where interpretability and computational speed are balanced.

Support Vector Machines (SVM) have also gained traction as robust ML tools for *S*_*w*_ prediction. Authors^34^ demonstrated SVM superior accuracy over ANN while developed a least-squares SVM (LS-SVM) variant that leveraged feature ranking to reduce input dimensionality without sacrificing performance. Comparative studies further validate SVM efficacy^35^ found SVM outperformed ANN, Random Forest (RF), and gradient boosting in limited-data sandstone reservoirs. Meanwhile, Authors^36^ evaluated ensemble methods (XGBoost, LightGBM, Adaboost, Catboost, Random Forest, and Super learner) in Russian sandstone reservoirs, with XGBoost marginally surpassing Super Learner models.

A notable contribution by authors^37^ optimized LS-SVM models for ϕ and *S*_*w*_ prediction in Norway’s Varg Field, identifying a minimal log suite - medium resistivity (*R*_*m*_), gamma ray (*GR*), caliper (*CALI*), and self-potential (*SP*) - as sufficient for maximal accuracy. Counterintuitively, expanding input variables degraded performance, suggesting parsimonious log selections may mitigate overfitting in data-constrained environments. A comprehensive literature survey about the applicability of machine learning in oil and gas exploration with emphasis on reservoir properties prediction discussed included in^37,38^

The reviewed literature highlights that most *ML* approaches for *S*_*w*_ prediction either rely on conventional, and often inaccurate, log-derived *S*_*w*_ for training, or depend on sparse and prohibitively expensive core data. This dependency represents a fundamental bottleneck in developing robust, field-scale ML models.

This research addresses this gap by proposing a novel, integrated two-stage framework. Our primary contribution is not simply the application of ML, but the 'optimization-first’ methodology that creates a high-fidelity, physics-informed training dataset from log data alone. First, the framework employs numerical optimization (e.g., Powell, Differential Evolution) to accurately determine a single, globally optimized Rw from a minimal log suite. Second, this robust *R*_*w*_ is used to generate a continuous, high-quality *S*_*w*_ log. This 'optimized-Sw’ log then serves as a 'pseudo-core’ target variable to train and rigorously evaluate a comprehensive suite of ML models, including advanced ensembles (XGBoost, CatBoost) and neural networks (ANNs, LSTM). This approach effectively bypasses the traditional reliance on sparse core analyses, representing the first systematic investigation to couple *R*_*w*_ optimization with advanced *ML* prediction for this purpose in shaley-sand formations. Furthermore, we rigorously validate the robustness and generalizability of this framework on two distinct datasets: a primary dataset from the Norwegian North Sea and a blind-test dataset from the Egyptian Western Desert. By demonstrating consistent accuracy across different basins, this study advances a universally adaptable and cost-effective solution for Sw estimation, bridging the gap between ML innovation and operational petrophysical workflows.

## Material and methodology

### Dataset description

This study utilizes a total of 11 well logs (Table 1), comprising nine wells from the Norwegian North Sea offshore region, and two additional wells from the Egyptian Western Desert, situated in the Abu El-Gharadig and Matruh - Shushan basins. The Norwegian dataset is sourced from the publicly available Geolink Dataset^39^, licensed under NOLD 2.0, and provided by *Geolink AS* company*.* Complementary sample and cores analyses for the Norwegian wells were derived from the Norwegian Petroleum Directorate (NPD) fact pages^40^.

**Table 1:** Geographic distribution, basin affiliation, and key parameters of the wells included in this study.

Well No.	Location/Basin	Basin/Sub-basin	Formation (*Age*)	Depth interval (*m*)	Rw^*^ (*Ω·m*)	Data source
NS-01	Norwegian North Sea	Southern Viking Graben	Sleipner and Hugin (*M. Jurassic*)	2985 - 3045	0.047	Geolink and factpages
NS-02	Norwegian North Sea	Southern Viking Graben	Sleipner and Hugin (*M. Jurassic*)	2895 - 2936	0.045	Geolink and factpages
NS-03	Norwegian North Sea	Southern Viking Graben	Hugin (*M. Jurassic*)	2384 - 2430	0.05	Geolink and factpages
NS-04	Norwegian North Sea	Southern Viking Graben	Sleipner and Hugin (*M. Jurassic*)	3342 - 3490	0.051	Geolink and factpages
NS-06	Norwegian North Sea	Southern Viking Graben	Hugin (*M. Jurassic*)	2278 - 2505	0.015	Geolink and factpages
NS-07	Norwegian North Sea	Southern Viking Graben	Heimdal (*Paleocene)*	2216 - 2160	0.05	Geolink and factpages
NS-08	Norwegian North Sea	Southern Viking Graben	Heimdal (*Paleocene)*	2068 - 2220	0.030	Geolink and factpages
NS-09	Norwegian North Sea	Southern Viking Graben	Sleipner (*M. Jurassic*) Statfjord (*E. Jurassic*)	3180 - 3310	0.045	Geolink and factpages
NST-05	Norwegian North Sea	Southern Viking Graben	Statfjord (*E. Jurassic*)	3695 - 3885	0.043	Geolink and factpages
EGT-01A	Egyptian Western Desert	Matruh - Shushan Basin	Upper and Lower Safa (Jurassic)	1519 - 1570	0.027	Proprietary Dataset
EGT-02B	Egyptian Western Desert	Abu El-Gharadig	Bahariya (u*pper cretaceous*)	2913 - 2953	0.038	Proprietary Dataset

* *R*_*w*_ Listed in the table along with lithology factors (a, m and n) are measured from samples and cores and utilized primarily for comparison with results obtained from current research.

### Rw estimation via optimization

This section details the systematic methodology developed to estimate *R*_*w*_ through optimization techniques. The framework integrates global and local optimization algorithms to minimize discrepancies between measured resistivity (*R*_*t*_) and Resistivity values derived from physics-based shaley-sand model^21^. The workflow is structured into three phases:

1. Data preparation.
2. Optimization framework.
3. Validation against conventional petrophysical models.

The methodology begins with log anomalies removal (De-spiking) and quality control to ensure robust input for optimization. Gamma-ray (GR) logs are utilized to calculate shale volume (*V*_*sh*_) using the following mathematical representation:

1 $$V_{sh} = \frac{{GR_{{{\mathrm{reading}}}} - GR_{{{\mathrm{clean}}}} }}{{GR_{{{\mathrm{shale}}}} - GR_{{{\mathrm{clean}}}} }}$$

Where $G{R}_{\mathrm{shale}} and G{R}_{\mathrm{clean}}$, obtained from log measurements from a thick unit of shale in the well of study calibrated to data from the Norwegian North Sea. Invalid entries are removed to exclude non-physical values. Neutron porosity and deep resistivity logs are retained as primary inputs.

The inverse problem is formulated to estimate global *R*_*w*_ and corresponding *S*_*w*_ that minimizes the difference between modeled and measured *R*_*t*_. The physics-based resistivity model integrates shale conductivity effects through a derivation from Schlumberger equation^21^:

2 $${R}_{t}=\frac{{\phi }^{2}}{0.2\cdot {R}_{w}\cdot \left(1-{V}_{sh}\right)\cdot \left({\left({S}_{w}\cdot \frac{{\phi }^{2}}{0.4\cdot {R}_{w}\cdot \left(1-{V}_{sh}\right)}\right)}^{2}+2\cdot {S}_{w}\cdot \frac{{\phi }^{2}}{0.4\cdot {R}_{w}\cdot \left(1-{V}_{sh}\right)}\cdot \frac{{V}_{sh}}{{R}_{sh}}\right)}$$

where *R*_*sh*_ denotes shale resistivity.

The objective function incorporates regularization to stabilize solutions:

3 $$Minimize f\left({s}_{w}^{ }, {R}_{w}^{ }\right)=\sqrt{\frac{1}{N}{\sum }_{i=1}^{N}{\left(R{t}_{\mathrm{meas}}^{\left(i\right)}-R{t}_{\mathrm{model}}^{\left(i\right)}\left({s}_{w}^{ }, {R}_{w}^{ }\right)\right)}^{2}} + \lambda \left({s}_{w}^{ 2}+{R}_{w}^{ 2}\right)$$

subject to physical constraints (based on regional analysis of *R*_*w*_ measurements from any nearby wells):

$$0 \le Sw \le 1, \hspace{1em}0.01 \le Rw \le 0.1 \hspace{0.17em}\Omega \cdot m$$

Where:

- *f(Sw,Rw):* The objective function to be minimized.
- *Rt*_meas_^(*i*)^: Measured deep resistivity at depth interval *i* (Ω⋅*m*).
- *Rt*_model_^(*i*)^ (*Sw*,*Rw*): Modeled resistivity at depth interval *i*, calculated using the Schlumberger shaley-sand equation (Equation 2).
- *N*: Total number of depth intervals (data points) in the log.
- *λ*: L2 regularization coefficient.

Four optimization algorithms were rigorously evaluated. They were selected for their complementary strengths to systematically compare the performance of different optimization philosophies:

- Powell and Nelder-Mead: These are high-efficiency, gradient-free local search (direct search) methods. They are well-suited for rapidly refining solutions in smooth, uni-modal problems and do not require derivatives.
- Differential Evolution (DE): This is a powerful, stochastic global optimization algorithm. It was chosen to robustly explore the entire search space and mitigate the risk of being trapped in a local minimum, which is common in complex geophysical inverse problems.
- COBYLA (Constrained Optimization BY Linear Approximation): This algorithm was specifically included for its ability to handle non-linear optimization problems with inequality constraints, which directly aligns with our problem’s need to bound Rw within physically realistic limits.

A concise summary of each method mathematical foundation is provided in Table 2, omitting detailed theoretical derivations to maintain focus on the applied framework. While acknowledging the mathematical complexity inherent to optimization techniques, this study prioritizes their practical implementation and comparative performance, as exhaustive algorithmic expansions fall beyond the scope of this reservoir characterization-focused investigation.

**Table 2:** Summary of optimization techniques employed in this study.

**Method**	**Mathematical formulation**	**Reference**
Powell	Iteratively minimizes along conjugate directions without derivatives: $x_{k + 1} = x_{k} + {\upalpha }_{k} d_{k}$	^41^
Nelder-Mead	Simplex-based direct search adjusting vertices via reflection (ρ), expansion (χ), contraction (γ), and shrinkage (σ): ${x}_{c}=\frac{1}{n}{\sum }_{i\ne h}{x}_{i}$	^42^
Differential evolution (DE)	Population-based global optimization with mutation, crossover, and selection: ${v}_{i}={x}_{r1}+F\left({x}_{r2}-{x}_{r3}\right)$ $${u}_{ij}=\left\{\begin{array}{c}{v}_{ij},\hspace{1em}{\mathrm{i}}{\mathrm{f}} \, {\mathrm{r}}{\mathrm{a}}{\mathrm{n}}{\mathrm{d}}\le CR\\ {\mathrm{x}}_{\mathrm{ij}},\hspace{1em}{\mathrm{o}}{\mathrm{t}}{\mathrm{h}}{\mathrm{e}}{\mathrm{r}}{\mathrm{w}}{\mathrm{i}}{\mathrm{s}}{\mathrm{e}}\end{array}\right.$$	^43^
COBYLA	Linear approximation of constraints: $\mathrm{min}f\left(x\right)\text{ s.t. }{c}_{i}\left(x\right)\le 0$	^44^

### Validation against conventional models

Results are benchmarked against Three industry-standard shaley-sand models:

1. Simandoux^13^:
  4 $${S}_{w}=\frac{a\cdot {R}_{w}}{2\cdot {\upphi }^{m}}\left[\sqrt{{\left(\frac{{V}_{sh}}{{R}_{sh}}\right)}^{2}+\frac{4\cdot {\upphi }^{m}}{a\cdot {R}_{w}\cdot {R}_{t}}}-\frac{{V}_{sh}}{{R}_{sh}}\right]$$
2. Schlumberger^21^:
  5 $${\mathrm{S}}_{\mathrm{w}}=\sqrt{\frac{{\left(\frac{{\mathrm{V}}_{\mathrm{sh}}}{{\mathrm{R}}_{\mathrm{sh}}}\right)}^{2}+\frac{{\upphi }^{2}}{0.2\cdot {\mathrm{R}}_{\mathrm{w}}\cdot {\mathrm{R}}_{\mathrm{t}}\cdot \left(1-{\mathrm{V}}_{\mathrm{sh}}\right)}-\frac{{\mathrm{V}}_{\mathrm{sh}}}{{\mathrm{R}}_{\mathrm{sh}}}}{\frac{{\upphi }^{2}}{0.4\cdot {\mathrm{R}}_{\mathrm{w}}\cdot \left(1-{\mathrm{V}}_{\mathrm{sh}}\right)}}}$$
3. Poupon and Leveaux (Indonesia)^14^:
  6 $${S}_{w}={\left(\frac{\sqrt{\frac{1}{{R}_{t}}}}{\frac{{V}_{sh}^{\left(1-0.5{V}_{sh}\right)}}{\sqrt{{R}_{sh}}}+\sqrt{\frac{{\upphi }^{m}}{a\cdot {R}_{w}}}}\right)}^\frac{2}{n}$$

Discrepancies between optimized and conventional *S*_*w*_-*R*_*w*_ pairs are quantified using root mean square error (RMSE) with the following equation:

7 $${\mathrm{RMSE}}=\sqrt{\frac{1}{N}{\sum }_{i=1}^{N}{\left({R}_{{t}_{\mathrm{meas}}}^{\left(i\right)}-{R}_{{t}_{\mathrm{model}}}^{\left(i\right)}\right)}^{2}}$$

Where:

- *N*: Total number of depth intervals or data points.
- *Rt*_meas_^(*i*)^: Measured resistivity at depth interval *i*.
- *Rt*_model_^(*i*)^: Modeled resistivity at depth interval i*i*, calculated using the Schlumberger shaley-sand equation.

### Dataset preparation

The integrity of petrophysical data forms the cornerstone of robust machine learning applications in reservoir characterization. This study adopts a systematic, multi-stage preprocessing pipeline to address inherent challenges in shaley sand formations, including data heterogeneity, measurement noise, and depth-dependent variability. The methodology encompasses five principal phases, each designed to enhance data quality while preserving geological realism.

#### Log quality control and initial processing

**Fig. 1:**
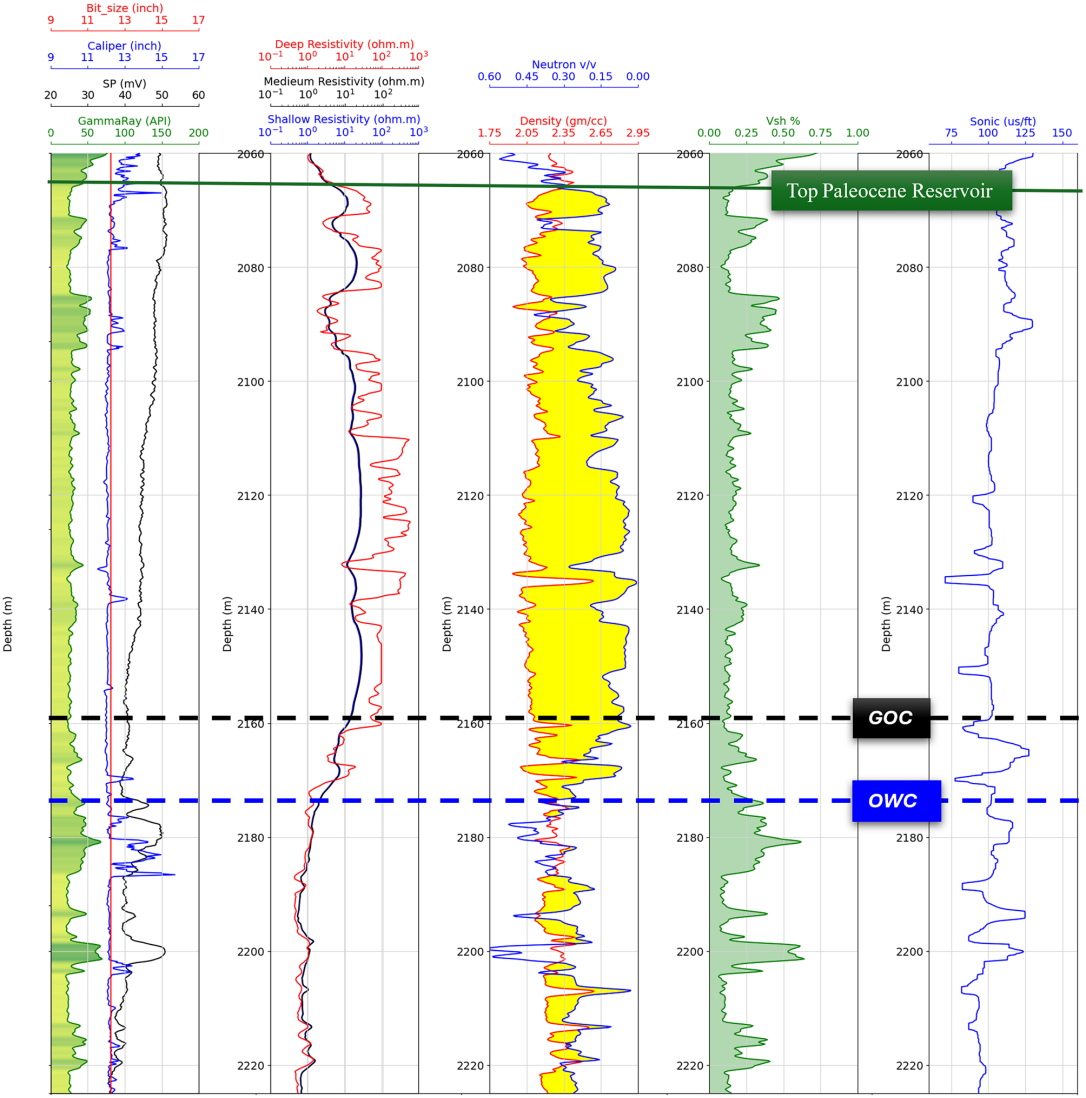
Log Display of well NS-08 showing Zone of interest (Top Paleocene Heimdal reservoir) fluid contacts. Gas-Oil-Contact (GOC) in Black dashed line, and Oil-Water-Contact (OWC) in blue dashed line.

The wireline dataset from twelve wells underwent rigorous quality assurance protocols. Initial processing included alignment of depth indices to a consistent sampling interval (0.1 m) to resolve discrepancies arising from tool stick-slip or borehole rugosity. Range validation excluded nonphysical values. Composite log displays were generated to visualize parameter distributions and detect Zones of interest, such as neutron-density crossover indicating porous reservoir formations (Fig. 1).

#### Handling missing data

Approximately 0.0012 % of the dataset exhibited missing values. Depths with any missing features were discarded to prevent any biased imputation.

#### Feature normalization

To minimize the impact of data fluctuations during the modeling process, and to avoid overfitting both input and output variables are normalized by the following equation:

8 $${n}{\prime}=\frac{n-{n}_{\mathrm{min}}}{{n}_{\mathrm{max}}-{n}_{\mathrm{min}}}$$

Where:

- $n$: the original value,
- ${n}_{\mathrm{min}}$: the minimum value in the dataset,
- ${n}_{\mathrm{max}}$: the maximum value in the dataset,
- ${n}{\prime}$: the normalized value scaled between 0 and 1.

**Fig. 2:**
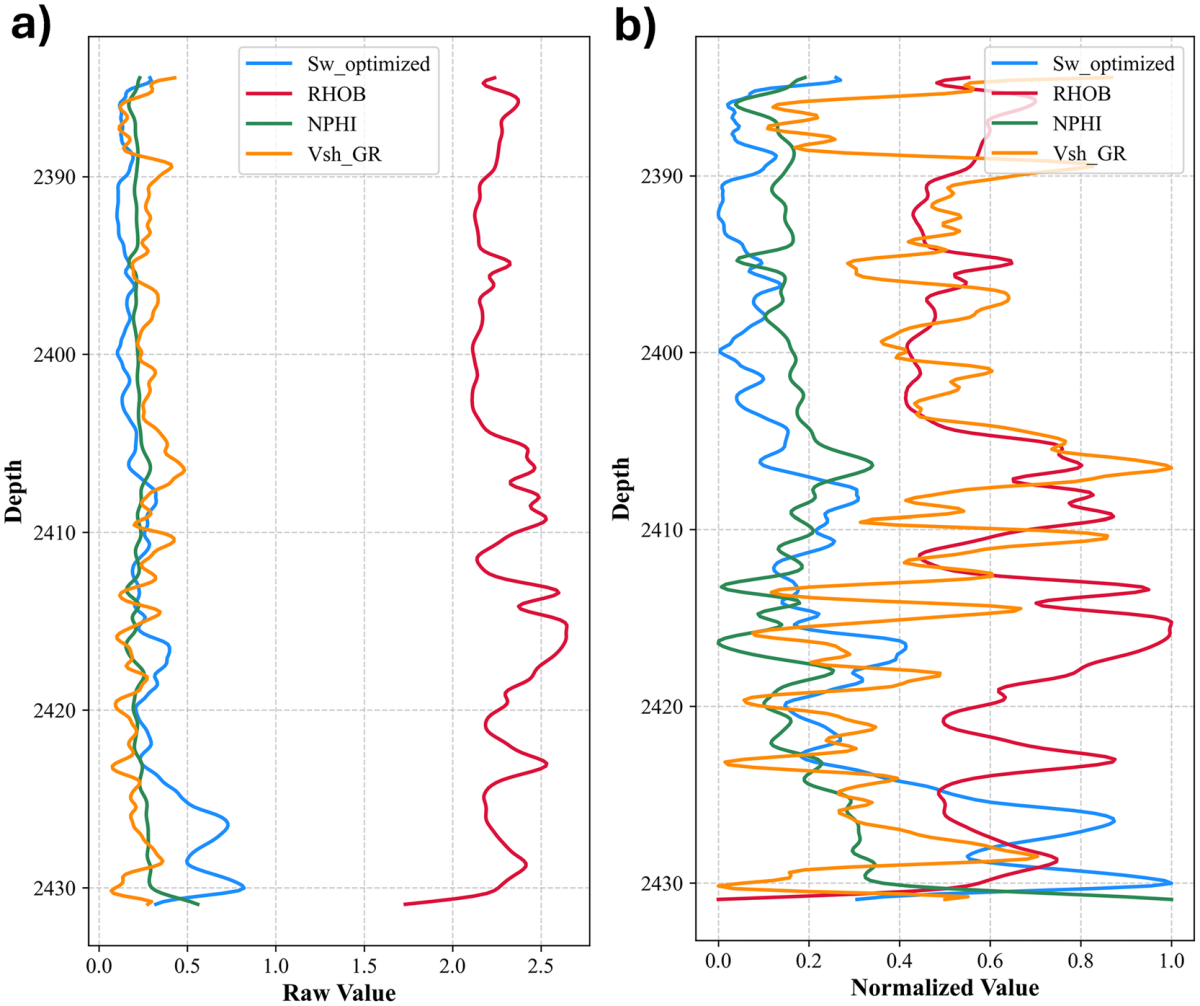
Raw and Normalized Petrophysical Logs vs. Depth for well NS-03. (**a**) Raw plots of versus depth (**b**) After applying normalization.

Figure 2 represents well NS-03 offering a dual perspective on petrophysical data: the raw data plot reveals distinct scaling differences among *S*_*w*__optimized, RHOB, NPHI, and *V*_*sh*_ across depth, highlighting inherent measurement variability and reservoir heterogeneity, while the normalized plot - achieved via scaling - facilitates a direct visual comparison by aligning the disparate logs onto a uniform [0,1] scale. This juxtaposition not only underscores the critical need for normalization in multi-variable reservoir analyses but also enhances interpretability by revealing subtle trends and correlations that are otherwise masked by scale differences, thereby supporting more informed reservoir characterization and integrated data interpretation.

#### Hybrid outlier detection framework

**Fig. 3:**
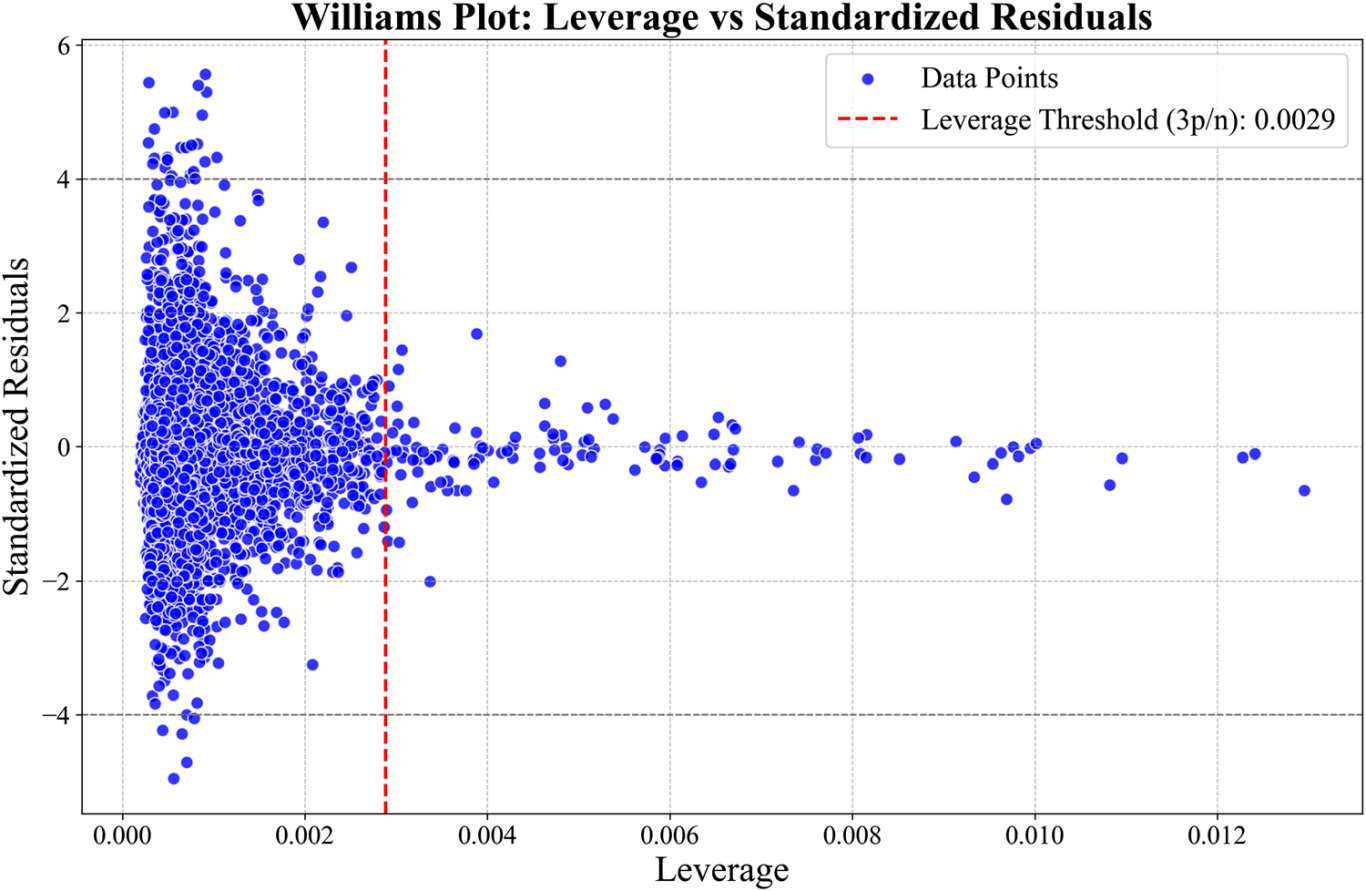
Delineation of potential outliers (Points within red zones) before further preprocessing the data for ML phase.

Outlier removal combined statistical leverage analysis with domain-specific constraints. The Williams plot methodology identified high-leverage points through hat matrix decomposition:

9 $$H=X{\left({X}^{T}X\right)}^{-1}{X}^{T}$$

where *X* represents the normalized feature matrix. Observations exceeding a *3(p/n*) leverage threshold (*p*=features, *n*=samples) were flagged. This threshold was not chosen randomly; it was determined after empirical testing of standard cutoffs (e.g., *2p/n, 4p/n, 5p/n*) and was found to provide the optimal balance between high-leverage outlier removal and data preservation for our subsequent ML models. These statistical outliers were further filtered through petrophysical bounds GR (10–180 API), NPHI (0.05–0.50.05.50), Rt (0.2–1000.2 $\Omega \cdot m$), and RHOB (1.9 to 2.96 gm/cc). This dual approach removed 3.2% of data, balancing noise reduction with sample retention (Fig.3).

#### Sensitivity analysis using relevancy factor and SHAP

To quantitatively assess the relative influence of petrophysical variables on the water saturation, a critical parameter in shaley-sand resistivity models, a sensitivity analysis was conducted using the relevancy factor (*r*). This statistical metric evaluates the linear correlation between input variables and the output (*n*), providing insight into parameter interdependencies that govern reservoir behavior.

The relevancy factor for each input variable x_*j*_ is computed as:

10 $${r}_{j}=\frac{{\sum }_{i=1}^{N}\left({x}_{j,i}-\overline{{x }_{j}}\right)\left({y}_{i}-\overline{y }\right)}{\sqrt{{\sum }_{i=1}^{N}{\left({x}_{j,i}-\overline{{x }_{j}}\right)}^{2}}\cdot \sqrt{{\sum }_{i=1}^{N}{\left({y}_{i}-\overline{y }\right)}^{2}}}$$

where:

- *xj*,*i*: Value of the *j*-th input variable at depth *i*.
- *yi*: Corresponding water saturation derived from optimization phase.
- $\overline{{x }_{j}}$, $\overline{y }$: Mean values of the *j*-th input and output, respectively.
- *N*: Total number of data points.

**Fig. 4:**
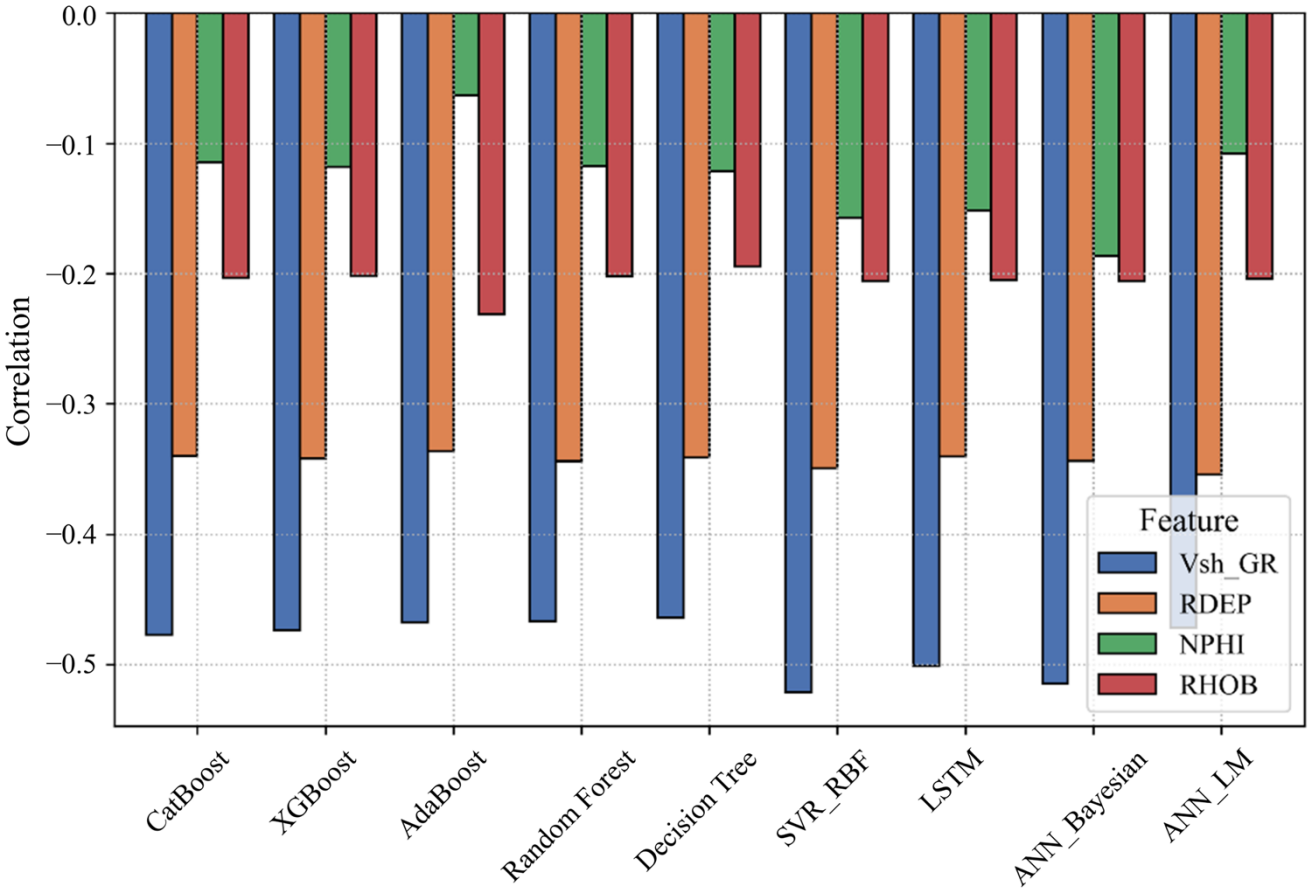
Relative impact of the well logs on *S*_*w*_ using relevancy factor (*r*) for adopted Machine learning algorithms.

The relevancy factor ranges from −1 (perfect inverse correlation) to +1 (perfect direct correlation), with magnitudes > 0.3 indicating significant relationships (Fig.4)

To move beyond simple linear correlations and gain a deeper, model-agnostic understanding of our trained models, we employed SHAP (SHapley Additive exPlanations). SHAP is a state-of-the-art method from cooperative game theory that provides robust, locally accurate, and consistent feature attributions. The core of SHAP is the Shapley value, which quantifies the contribution of each feature to a specific prediction by averaging its marginal contribution across all possible feature coalitions.

The Shapley value ${\upphi }_{i}$ for feature $i$ is formally defined as:

11 $${\upphi }_{i}\left(f,x\right)={\sum }_{S\subseteq F\setminus \{i\}}\frac{\left|S\right|!\left(\left|F\right|-\left|S\right|-1\right)!}{\left|F\right|!}\left[{f}_{x}\left(S\cup \{i\}\right)-{f}_{x}\left(S\right)\right]$$

where $F$ is the set of all features, $S$ is a subset of features, ${f}_{x}\left(S\right)$ is the model prediction using the feature values in $S$.

SHAP unites this concept with an additive feature attribution model, which explains a prediction ${f}_{x}$ as the sum of the base value ($E[f(z)]$) plus the SHAP values for each of the $M$ features:

12 $$f\left(x\right)=E\left[f\left(z\right)\right]+{\sum }_{i=1}^{M}{\upphi }_{i}\left(f,x\right)={\upphi }_{0}+{\sum }_{i=1}^{M}{\upphi }_{i}$$

**Fig. 5:**
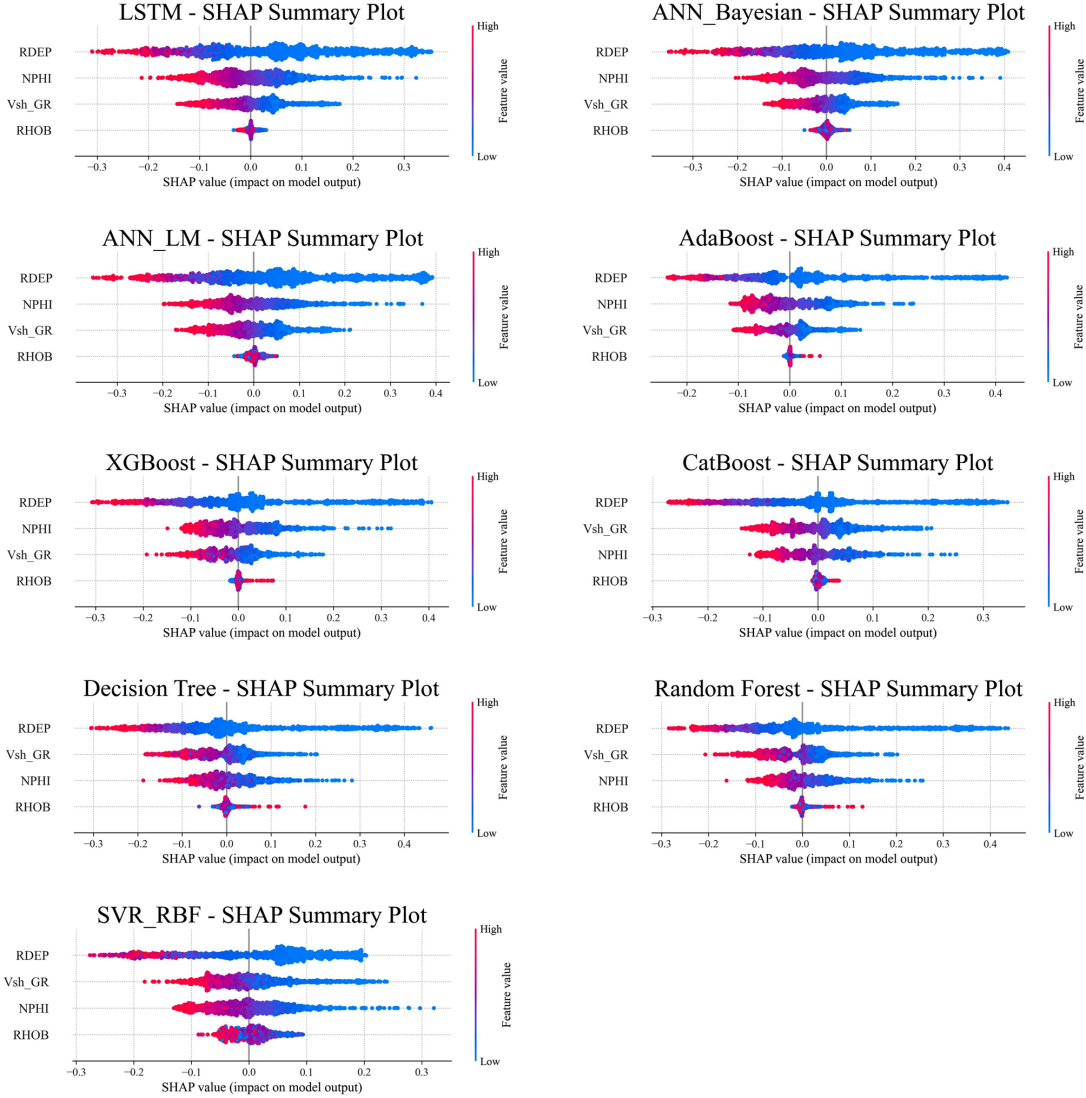
SHAP summary plots for the top-performing models. Features are ranked by global importance. The horizontal axis is the SHAP value (impact on output), and the color indicates the original feature value (*red=high*, *blue=low*), revealing the direction and magnitude of each feature influence.

The resulting SHAP summary plots (Fig. 5) providing two critical insights:

1. Global Feature Importance: Features ranked by their mean absolute SHAP value.
2. Local Feature Impact: A distribution visualizing the magnitude and direction (positive or negative) of each feature contribution to predicting water saturation.

### ML model training, refinement, and validation

The principal objective of this section is to delineate a systematic, data-driven framework for the development, validation, and refinement of ML models designed to predict *S*_*w*_ with high fidelity from well-derived datasets. This framework integrates critical phases of data preprocessing, algorithmic selection, performance benchmarking, and hyperparameter optimization - each structured to ensure the derivation of models that are both statistically robust and operationally generalizable for subsurface reservoir assessment.

**Fig. 6:**
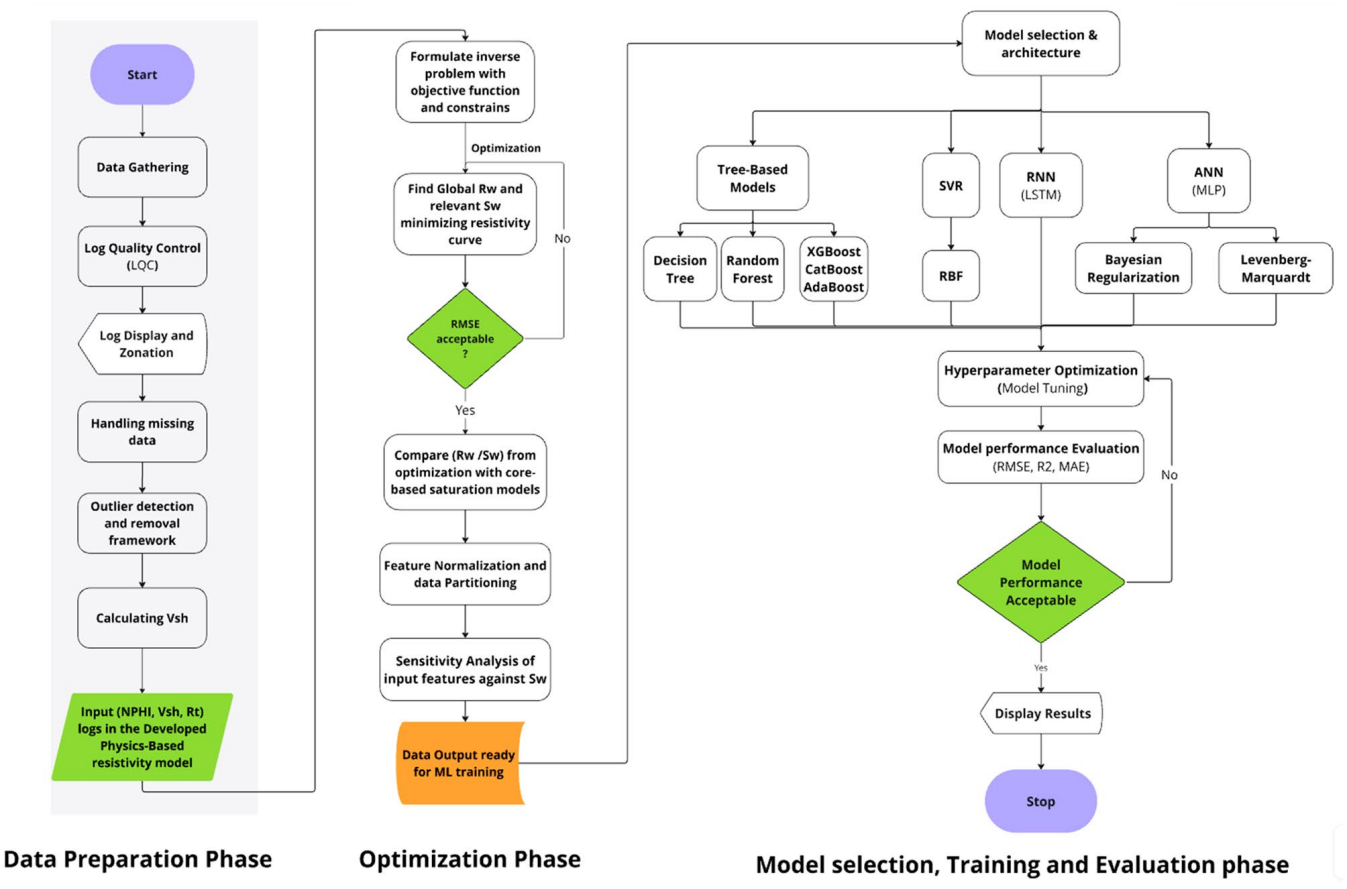
Workflow schematic for the automated estimation of *R*_*w*_ and *S*_*w*_ prediction.

Summarized ML algorithms in Table 3 when appropriately selected and rigorously optimized, exhibit the capacity to discern intricate, non-linear relationships within high-dimensional and noise-contaminated datasets, thereby offering transformative potential for reservoir characterization. The efficacy of such models, however, is contingent upon a methodologically sound modeling pipeline. This necessitates judicious algorithm selection aligned with data characteristics, the application of stringent validation protocols, and systematic hyperparameter optimization to maximize predictive performance while mitigating overfitting. The entire framework for *R*_*w*_ estimation alongside *S*_*w*_ prediction integrates advanced machine learning with rigorous validation protocols, as outlined in Figure 6.

**Table 3:** Comparative summary for ML algorithms implemented in the current study.

**Model**	**Description**	**Mathematical principle**	**Mathematical formulation & key concepts**
**Decision Tree (DT)**	Hierarchical, rule-based model splitting features at optimal thresholds^60^	Recursive binary splitting to minimize MSE by choosing the best split that reduces variance in target values.	Splits features to minimize impurity: ${\mathrm{MSE}}=\frac{1}{n}{\sum }_{i=1}^{n}{\left({y}_{i}-\widehat{{y}_{i}}\right)}^{2}$
**Random Forest (RF)**	An ensemble of decision trees using bagging and feature randomness^48^	Reduces overfitting by averaging predictions from many uncorrelated trees trained on bootstrapped samples.	Averaging predictions: $\widehat{{f}_{RF}}\left(x\right)=\frac{1}{T}{\sum }_{t=1}^{T}\widehat{{f}^{\left(t\right)}}\left(x\right)$
**AdaBoost (AB)**	Sequential ensemble where each model corrects predecessor’s errors^49^	Emphasize difficult samples by assigning them higher weights, improving weak learners.	Weighted voting: $\widehat{y}={\sum }_{m=1}^{M}{\alpha }_{m}{h}_{m}\left(x\right)$ ${\alpha }_{m}=\frac{1}{2}\mathrm{ln}\left(\frac{1-{\epsilon }_{m}}{{\epsilon }_{m}}\right)$
**XGBoost (XGB)**	Gradient boosting with regularization and shrinkage^46^	Optimizes a regularized loss function, improving generalization.	Additive model: $\widehat{{y}_{i}}={\sum }_{k=1}^{K}{f}_{k}\left({x}_{i}\right),\hspace{1em}{f}_{k}\in \mathcal{F}$ Objective: ${\mathrm{Obj}}=\sum l\left({y}_{i},\widehat{{y}_{i}}\right)+\sum\Omega \left({f}_{k}\right)$ $\Omega \left(f\right)=\gamma T+\frac{1}{2}\lambda |w{|}^{2}$
**CatBoost (CB)**	Categorical boosting with ordered boosting to avoid overfitting^47^	Handles categorical variables using permutations and target statistics.	Uses ordered statistics to prevent target leakage; minimizes: $L=\sum {\left({y}_{i}-\widehat{{y}_{i}}\right)}^{2}+{\mathrm{Reg}}$
**SVR (RBF Kernel)**	Regression model that minimizes error within an epsilon-insensitive tube^51^	Uses kernel trick to fit nonlinear functions in high-dimensional space.	Optimization objective: $min\frac{1}{2}{w}^{T}w+C{\sum }_{i}\left[\mathrm{max}\left(0,\left|{y}_{i}-f\left({x}_{i}\right)\right|-\varepsilon \right)\right]$ $K\left(x,{x}{\prime}\right)=\mathrm{exp}\left(-\gamma |x-{x}{\prime}{|}^{2}\right)$
**Feedforward ANN (Bayesian Regularization)**	Fully connected layers for regression tasks^54^	Learns weights to minimize error and avoid overfitting using Bayesian probability	Posterior over weights: $P\left( {w\left| D \right.} \right) \propto \exp \left( { - \frac{1}{{2\sigma^{2} }}\sum \left( {y_{i} - \widehat{{y_{i} }}} \right)^{2} - \frac{\lambda }{2}\sum w_{j}^{2} } \right)$
**ANN (Levenberg-Marquardt)**	Fully connected layers for regression tasks^55^	Learn weights to minimize error and avoid overfitting using Levenberg- Marquardt optimization.	Optimization via second-order update: ${w}_{\mathrm{new}}=w-{\left({J}^{T}J+\mu I\right)}^{-1}{J}^{T}e$
**LSTM**	Recurrent neural network with memory gates for sequential data^57^	Maintains long-term memory via gated cell structures (forget, input, output gates).	Memory cell and gates: Forget gate: ${f}_{t}=\sigma \left({W}_{f}\left[{h}_{t-1},{x}_{t}\right]+{b}_{f}\right)$ Input gate: ${i}_{t}=\sigma \left({W}_{i}\left[{h}_{t-1},{x}_{t}\right]+{b}_{i}\right)$ State: ${C}_{t}={f}_{t}*{C}_{t-1}+{i}_{t}*\widetilde{{C}_{t}}$

The preprocessed dataset, comprising data from eight distinct wells in the North Sea (*NS-01* through *NS-09*), was designated solely for the training and cross-validation phase. A comprehensive 5-fold cross-validation protocol was implemented on this entire pooled training set to rigorously assess model performance and generalizability. The three remaining wells (*NST-05* from Norway, *EGT-01A* and *EGT-02B* from Egypt) were held out as a completely separate blind test dataset. These wells were not used in any aspect of training, validation, or hyperparameter tuning, and were used only once to evaluate the final, ability of the trained models to generalize to unseen data and geologically distinct basins.

**Fig. 7:**
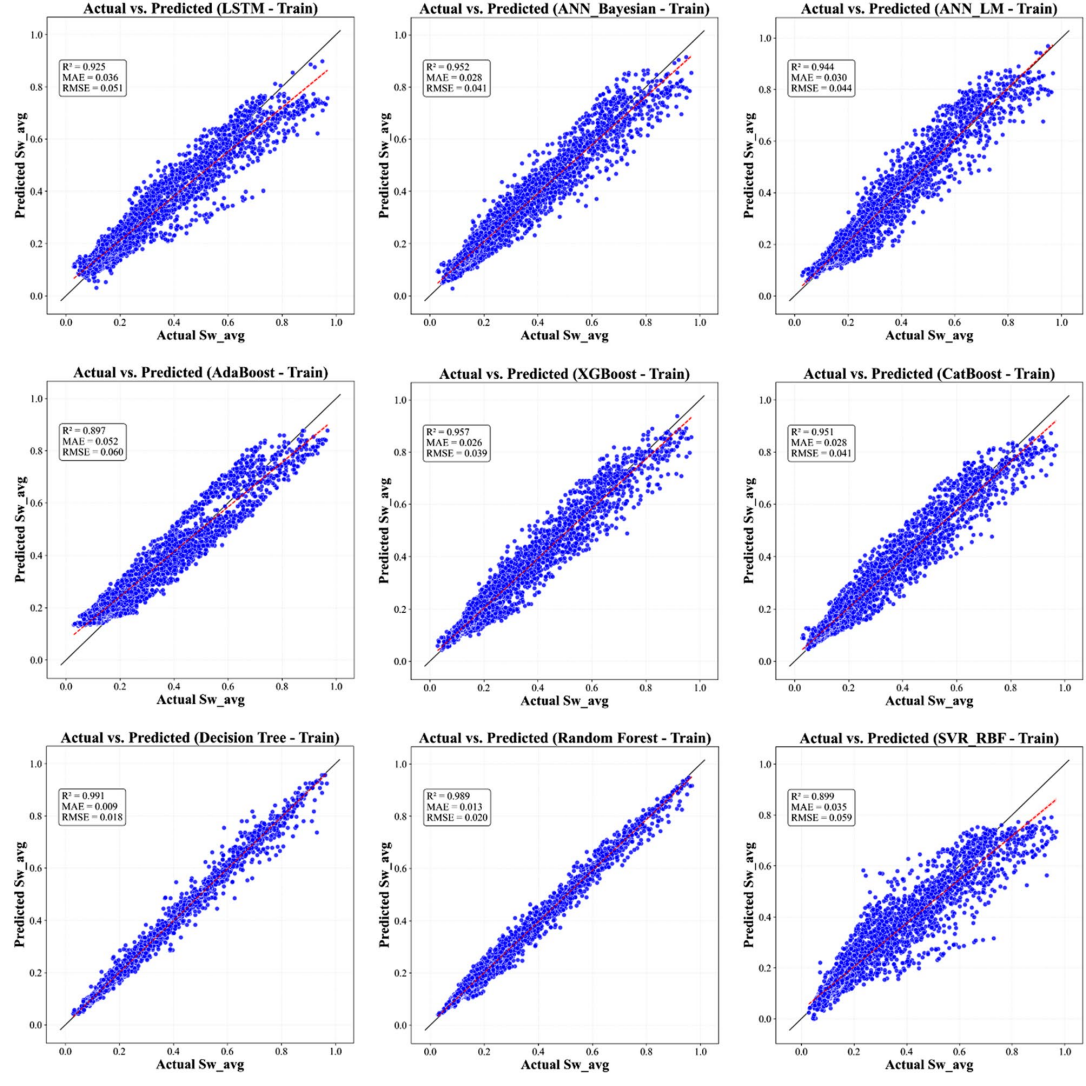
Actual versus predicted *S*_*w*_ from All ML Models - Training Phase.

**Fig. 8:**
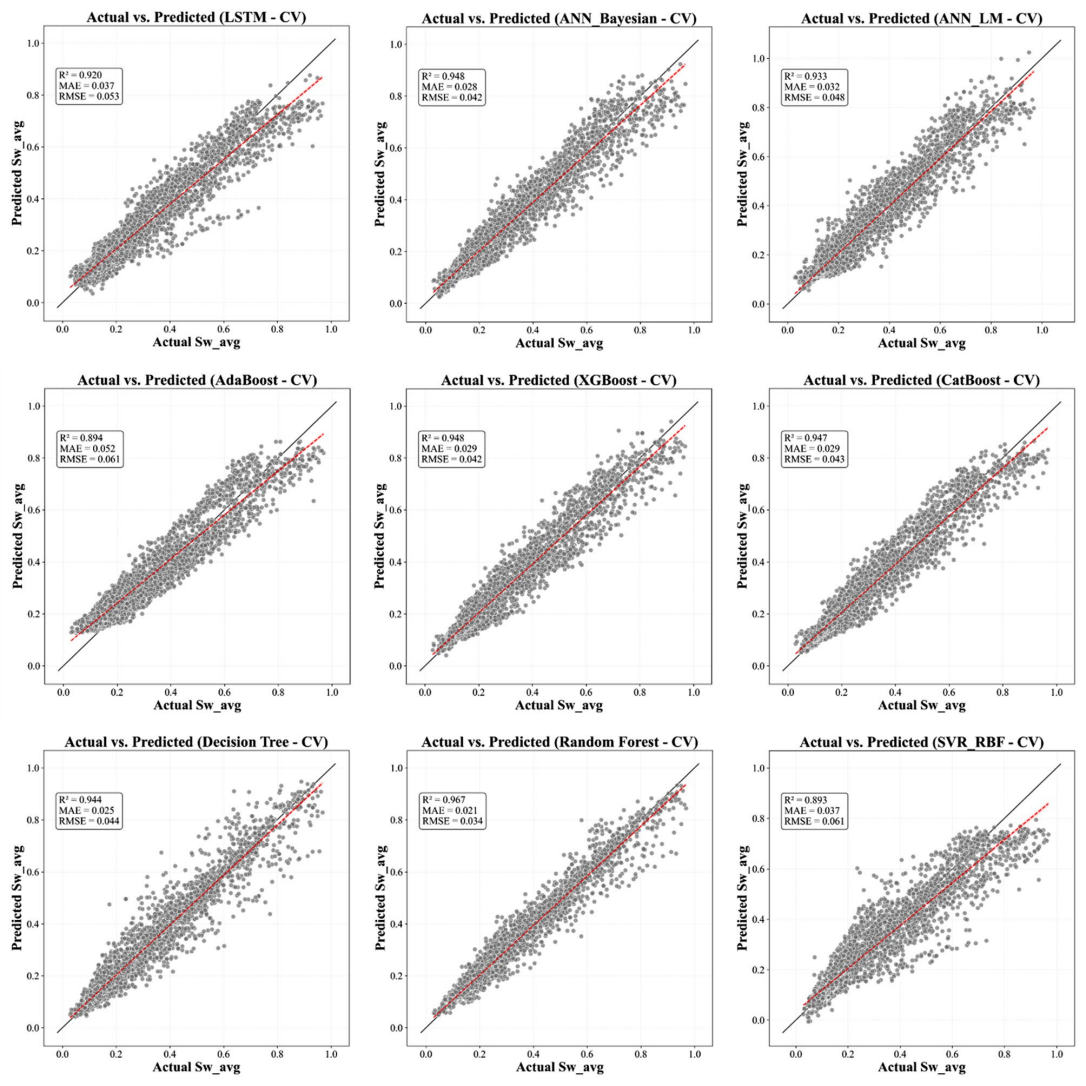
Actual versus predicted S_w_ from All ML Models - Validation Phase.

**Fig. 9:**
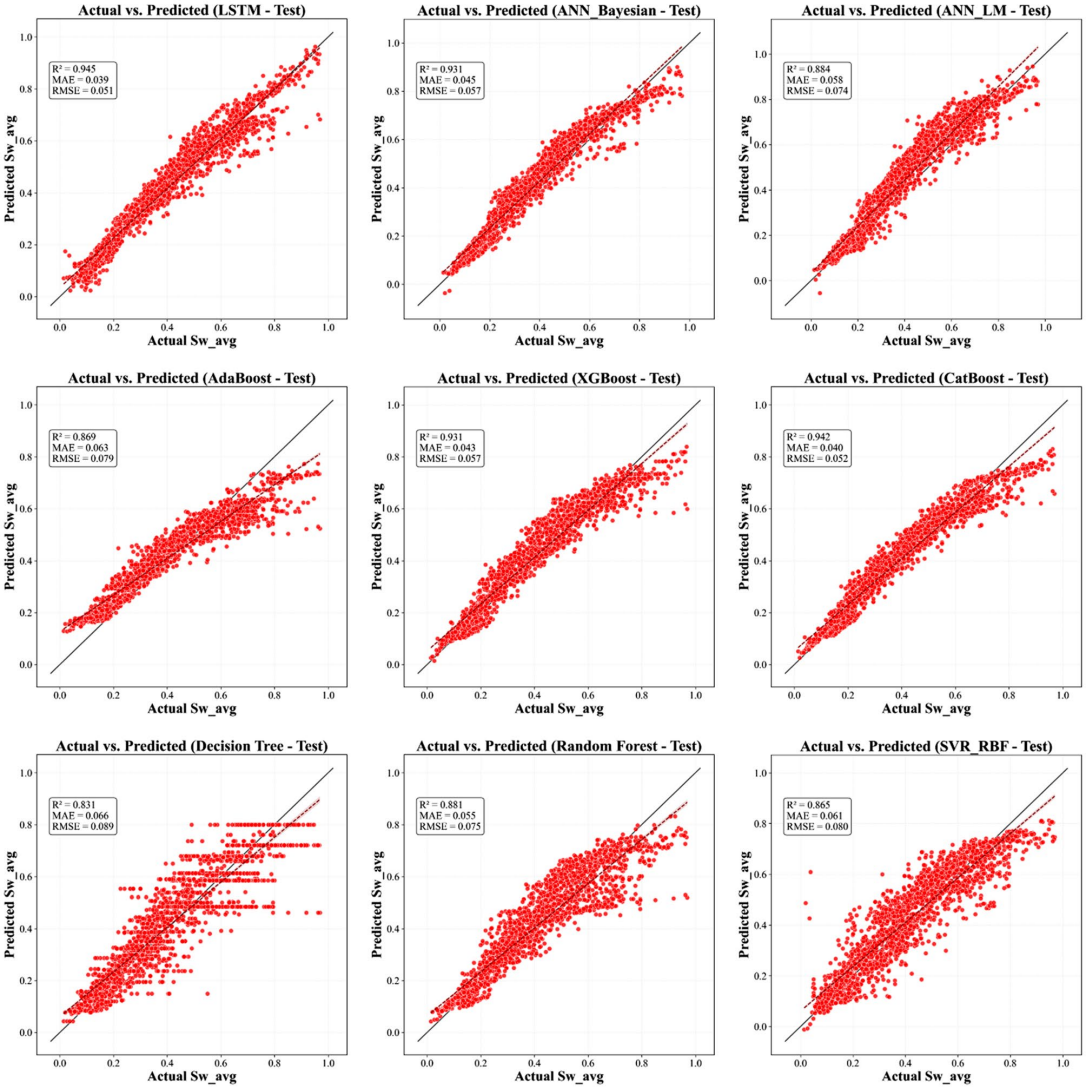
Actual versus predicted S_w_ from All ML Models - Testing Phase.

This section methodically articulates the architecture of the proposed pipeline, structured as follows:

1. Evaluates a suite of supervised learning algorithms, encompassing Tree-based ensemble methods (e.g., XGBoost, AdaBoost, CatBoost, Decision Tree, and Random Forest) alongside regularized regression techniques, to benchmark predictive accuracy and performance efficiency (Figures. 7, 8 and 9).
2. Employs multi-metric evaluation - including root mean square error (*RMSE*), mean absolute error (*MAE*), and coefficient of determination (R^2^) - to quantify model fidelity and guide algorithm selection.
3. Implements combinatorial search strategies, such as grid search and randomized cross-validation, to identify model configurations that optimally balance bias-variance trade-offs.

The algorithmic selection for this investigation was guided by rigorous evaluation of machine learning techniques with demonstrated efficacy in addressing the characteristic challenges of geophysical datasets - including non-linear parameter interactions, high-dimensional feature spaces, and heteroscedastic data structures. The following methodologies were prioritized based on their theoretical compatibility with petrophysical prediction tasks and empirical performance in analogous regression applications:

1. Tree-Based Ensemble Methods (Random Forest, Decision Trees, XGBoost, CatBoost, AdaBoost):

Exhibit inherent noise tolerance through bootstrap aggregation and stochastic subspace sampling, coupled with interpretability via feature significance metrics. Their hierarchical decision boundaries enable robust modeling of non-linear relationships in tabular petrophysical data while mitigating overfitting through ensemble variance reduction.

2. Support Vector Regression (SVR):

Kernel-based transformations (e.g., radial basis functions) facilitate high-dimensional hyperplane construction for continuous *S*_*w*_ prediction, particularly effective in complex, non-convex solution spaces where linear separability is absent. Theoretical guarantees on generalization error bounds enhance reliability in sparse-data regimes.

3. Artificial Neural Networks (ANNs):

**Feedforward Architectures**: Leverage multi-layer perceptron (*MLP*) to model hierarchical feature representations, excelling in capturing deep non-linearities between static well log variables and *S*_*w*_.

**Long Short-Term Memory (LSTM) Networks**: Specialized for sequential data analysis, these architectures were included to decode spatial dependencies in the depth-indexed logging measurements. Our rationale is that geological properties are not independent point-to-point but follow vertical depositional sequences (e.g., fining-upward or coarsening-upward cycles). We hypothesized an LSTM may capture these spatiotemporal patterns, which a standard feedforward ANN would miss. This approach is supported by a growing body of literature where LSTMs have been successfully applied to decode sequential dependencies in well-log data for tasks such as synthetic log generation, lithology identification, and missing log prediction In the development of machine learning models for predicting *S*_*w*_ from well log data, a suite of optimization methodologies was rigorously implemented, each tailored to the architectural and functional characteristics of the respective algorithms.

For gradient-boosted ensemble architectures (XGBoost, CatBoost), sequential additive learning frameworks were employed, wherein successive decision trees were iteratively constructed to minimize residual errors from prior ensemble iterations^45^. To regulate model complexity and counteract overfitting, these algorithms incorporated shrinkage mechanisms (i.e., learning rate attenuation), which attenuate the contribution of individual trees during gradient updates^46^. Both models further integrated early stopping protocols, whereby training was halted upon stagnation of validation loss improvement over predefined epochs, thereby enhancing computational efficiency while preserving generalization capacity^47^. For Random Forest and AdaBoost, bootstrap aggregation (bagging) and adaptive reweighting of misclassified samples were implemented to reduce variance and bias, respectively^48,49^^.^

The SVR framework adopted an ε-insensitive loss function, which tolerates residual errors within a margin of ε, thereby prioritizing corrections for deviations exceeding this threshold^50^. Optimization entailed solving a convex quadratic programming problem to minimize a regularized objective function, balancing a penalty term (governed by hyperparameter *C*) against the ε-insensitive loss^51^. This formulation ensures a principled trade-off between model complexity and error tolerance, rendering SVR particularly adept at capturing nonlinear relationships in high-dimensional feature spaces^52^.

For neural network-based models - specifically the Long Short-Term Memory (*LSTM*) network and two variants of feedforward Artificial Neural Networks (*ANNs*) optimized using Bayesian Regularization (*BR*) and the Levenberg-Marquardt (*LM*) algorithm - gradient-based optimization formed the backbone of the training process. Central to this was the backpropagation algorithm^53^, which computed the gradients of the loss function with respect to the network weights, thereby enabling iterative parameter updates through variants of gradient descent to minimize the overall prediction error.

In the Bayesian Regularization (*BR*) framework, the standard error-based loss function was augmented with a Tikhonov regularization term that penalized large weight magnitudes. This not only mitigated overfitting but also provided a probabilistic interpretation of model complexity, promoting smoother and more generalizable solutions by effectively balancing data fidelity and model simplicity^54^. In the ANN-LM architecture, the Levenberg-Marquardt (*LM*) optimization algorithm was employed as a robust second-order method that combined the curvature-sensitive Gauss-Newton approximation with the stability of gradient descent. This hybrid strategy was particularly advantageous for small-to-medium sized nonlinear regression problems, offering faster convergence and superior stability compared to traditional first-order methods^55^.

The LSTM network incorporated stochastic dropout regularization with a dropout rate during training, randomly deactivating a subset of neurons in each iteration. This method effectively reduced the risk of co-adaptation among neurons, thereby enhancing the model’s ability to generalize to unseen data^56^. Additionally, activation functions were carefully selected based on empirical performance and architectural demands: the hyperbolic tangent (tanh) activation function was employed in the recurrent layers to support stable gradient flow over long temporal sequences, which is critical for capturing sequential dependencies^57^; conversely, Rectified Linear Units (ReLU) were used in the feedforward layers due to their non-saturating nature, which promotes efficient training and alleviates vanishing gradient problems^58^.

The preprocessed dataset, comprising data from eight distinct wells in the North Sea, was designated solely for the training phase of the modeling process. During this phase, a comprehensive 5-fold cross-validation protocol was implemented to rigorously assess and improve model performance, ensuring the generalizability and robustness of the learned relationships. Within each fold, a further internal partitioning of 20% of the training data was utilized during hyperparameter tuning of deep learning architectures - such as LSTM and ANN models - to fine-tune model parameters and mitigate overfitting. This division and validation strategy provided iterative feedback on model accuracy across diverse subsets of the training set.

Hyperparameter optimization is a critical component of the proposed framework, directly influencing the predictive accuracy and generalizability of the machine learning models. In our study, a systematic and rigorous hyperparameter tuning strategy was implemented using both grid search and randomized cross-validation approaches (Table 4). This strategy enabled a comprehensive exploration of the hyperparameter space encompassing parameters such as the number of estimators, maximum tree depth, learning rate, regularization coefficients, and dropout rates to achieve an optimal balance between model complexity and generalization performance. For neural network architecture, advanced techniques including Bayesian Regularization and the Levenberg-Marquardt algorithm were leveraged alongside stochastic dropout and early stopping criteria based on validation loss. These measures not only mitigated the risk of overfitting but also enhanced the stability and convergence speed during training. Collectively, the fine-tuned hyperparameters, as delineated in Table 5, underpin the robust performance observed across the training, validation, and test phases, cementing the framework efficacy in predicting water saturation in shaley-sand reservoirs with high precision^59^

**Table 4:** Hyperparameter search space for machine learning models.

**Hyperparameter**	**XGBoost**	**Random Forest**	**SVR (RBF)**	**CatBoost**	**AdaBoost**	**Decision Tree**
Tuning Method	Grid Search	Randomized Search (20 iter)	Grid Search	Grid Search	Grid Search	Grid Search
n_estimators	[50, 100, 150]	[50, 100, 150, 200]			[50, 100, 150]	
iterations				[50, 100, 150]		
max_depth	[3, 5, 7]	[None, 5, 10, 15]			Fixed at 4*	[None, 3, 5, 7]
depth				[3, 4, 5]		
learning_rate	[0.01, 0.1, 0.3]			[0.05, 0.1, 0.15]		
min_samples_split		[2, 4, 6]				[2, 4, 6]
min_samples_leaf		[1, 2, 3]				[1, 2, 3]
C			[0.1, 1, 10, 100]			
epsilon			[0.01, 0.1, 0.5]			
gamma			[‘scale’, ‘auto’]			

**Hyperparameter**	**LSTM**	**ANN_LM**	**ANN_Bayesian**
Tuning Method	Keras Tuner (Random Search)	Keras Tuner (Random Search)	Keras Tuner (Random Search)
units	Integer: 20 to 60		
dropout	Float: 0.1 to 0.5		
units1		Integer: 32 to 128	Integer: 16 to 48
units2		Integer: 16 to 64	Integer: 8 to 32
dropout		Float: 0.1 to 0.5	
LSTM_LR	Log-uniform: 1×10^−4^ to ×10^−2^		
ANN_LR		Log-uniform: 1×10^−4^ to ×10^−2^	Log-uniform: 1×10^−4^ to ×10^−2^

**Table 5:** Hyperparameters settings for each ML Algorithm.

**Model**	**Number of Estimators**	**Maximum Depth**	**Learning** **rate**	**Kernel**	**C**	**epsilon**	**gamma**	**Hidden Layers**	**Neurons per Layer**	**Activation**	**Epochs**
XGBoost	200	10	0.05								
Random Forest	150	15									
CatBoost	100	10	0.1								
AdaBoost	100		0.05								
SVR (RBF)				RBF	100	0.1	0.01				
LSTM								1 LSTM + Dense	50	Tanh + Linear	100
ANN (BR)								2	20, 10	ReLU	200
ANN (LM)								3	30, 15, 10	Tanh	150

To ensure optimal performance and mitigate overfitting, the hyperparameters for each machine learning model were systematically optimized using a 5-fold cross-validation (CV) strategy. The search was guided by maximizing the R^2^ score. We employed *GridSearchCV* for models with smaller search spaces and *RandomizedSearchCV* (Keras Tuner) for larger spaces to balance computational cost and search breadth and presented.

A 5-fold cross-validation protocol was implemented. In our current study, we used a single fixed *random_state=42* for our 5-fold cross-validation strikes an optimal balance between computational efficiency and bias-variance trade-offs, reducing dependence on a single train-test partition^61^. This was an intentional choice to ensure that all models were trained and validated on the exact same 5 folds, allowing for a fair and reproducible comparison. This approach, coupled with early stopping and hyperparameter tuning, ensures that model performance reflects true generalizability rather than overfitting idiosyncratic data configurations^62^.

### Evaluation metrics

To evaluate the predictive performance of the regression models, three established metrics were employed: Mean Absolute Error (MAE), Root Mean Squared Error (RMSE), and the Coefficient of Determination (*R*^2^). These metrics collectively assess accuracy, error sensitivity, and explanatory power, addressing distinct facets of model performance.

#### Mean Absolute Error (MAE)

The MAE quantifies the average magnitude of absolute deviations between predicted ($\widehat{{y}_{i}}$) and observed (${y}_{i}$) values, computed as:

13 $${\mathrm{MAE}}=\frac{1}{n}{\sum }_{i=1}^{n}\left|{y}_{i}-\widehat{{y}_{i}}\right|$$

where *n* denotes the total number of observations. MAE is inherently interpretable and robust to scale, though it imparts equal weight to all residuals, potentially underemphasizing the influence of extreme outliers^63^.

#### Root Mean Squared Error (RMSE)

RMSE penalizes larger errors quadratically, offering sensitivity to outliers, and is defined as:

14 $${\mathrm{RMSE}}=\sqrt{\frac{1}{n}{\sum }_{i=1}^{n}{\left({y}_{i}-\widehat{{y}_{i}}\right)}^{2}}$$

This metric is particularly advantageous in contexts where substantial prediction errors carry disproportionately higher operational or financial risks^64^.

#### Coefficient of determination (${R}^{2}$)

${R}^{2}$ evaluates the proportion of variance in the dependent variable explained by the model, expressed as:

15 $${R}^{2}=1-\frac{{\sum }_{i=1}^{n}{\left({y}_{i}-\widehat{{y}_{i}}\right)}^{2}}{{\sum }_{i=1}^{n}{\left({y}_{i}-\overline{y }\right)}^{2}}$$

where $\overline{y }$​ represents the mean of observed values. Values approaching 1 indicate near-complete explanatory capability, while values near 0 suggest the model performs comparably to a naïve mean predictor^65^.

#### Statistical significance via paired T-Test

To rigorously validate whether the observed differences in performance between our top-performing machine learning models were statistically significant or merely the result of random chance, we employed a paired-sample t-test. This statistical test is specifically suited for comparing two models because their performance metrics (*RMSE*) were derived from the same 5-fold cross-validation dataset. The “paired” nature of the test arises from the fact that both models were trained and validated on the exact same five data splits (folds), allowing for a direct, fold-by-fold comparison of their errors.

The test is centered on analyzing the differences in the performance scores (in our case, the *RMSE*) for each fold. Let ${D}_{i}$ be the difference in RMSE between Model 1 and Model 2 on the $i$-th fold:

16 $${D}_{i}={\mathrm{RMSE}}_{\text{Model 1, fold }i}-{\mathrm{RMSE}}_{\text{Model 2, fold }i}$$

Our primary goal was to test the null hypothesis (H_0_), which states that there is no mean difference in performance between the two models. The alternative hypothesis (${H}_{a}$) states that a significant difference exists.

- Null Hypothesis (${H}_{0}$): ${\upmu }_{D}=0$ (The mean of the differences is zero).
- Alternative Hypothesis (${H}_{a}$): ${\upmu }_{D}\ne 0$ (The mean of the differences is not zero).

The t-statistic is calculated as the mean of the differences ($\overline{D })$ divided by its standard error ($S{E}_{D}$):

17 $$t=\frac{\overline{D }-{\upmu }_{0}}{S{E}_{D}}=\frac{\overline{D}}{{s }_{D}/\sqrt{n}}$$

where:

- $\overline{D }$ is the sample mean of the differences ${D}_{i}$ across the $n$ folds.
- $n$ is the number of folds (in our case, $n$ =5).
- ${s}_{D}$ is the sample standard deviation of the differences.
- ${\upmu }_{0}$ is the hypothesized population mean difference (0 under the null hypothesis).

From this t-statistic, a p-value is computed. This p-value represents the probability of observing our results (or more extreme) if the null hypothesis were true. In line with conventional scientific practice, we used a significance level ${(}\alpha {)}$ of 0.05. A p-value less than 0.05 indicates that the observed difference in model performance is statistically significant, allowing us to reject the null hypothesis and conclude that the models’ performances are genuinely different.

### Computational methods and software

The entire data processing, petrophysical optimization, and machine learning workflow was conducted using the *Python 3.11.9* programming language and its scientific computing ecosystem. Numerical computation and data structuring were performed using *NumPy* and *Pandas*, respectively. The primary machine learning framework was *Scikit-learn*, which was utilized for data preprocessing (e.g., *MinMaxScaler*), model cross-validation (*KFold*, *GridSearchCV*, *RandomizedSearchCV*), and evaluation (*r2_score, mean_absolute_error*). We implemented classical models including *RandomForestRegressor*, *AdaBoostRegressor*, *DecisionTreeRegressor*, and *SVR* from *Scikit-learn*, supplemented by specialized gradient boosting libraries *XGBoost* and *CatBoost*. Deep learning models (*ANN* and *LSTM*) were constructed using the *TensorFlow* framework with its high-level *Keras* API. The *Scikeras* library was employed as a wrapper to ensure *Scikit-learn* compatibility for the *Keras* models, and *Keras Tuner* was used for neural network hyperparameter optimization. Model interpretability was assessed using *SHAP*. Statistical significance was tested using the paired t-test function (*ttest_rel*) from *SciPy*. All visualizations were generated using *Matplotlib* and *Seaborn.*

## Results and discussion

### Optimization results

The results of *R*_*w*_ estimation via our advanced optimization framework demonstrate the efficacy of the proposed methodology in reconciling the inherent complexities of shaley-sand reservoirs. Four optimization algorithms - Powell, Nelder-Mead, Differential Evolution, and COBYLA - were evaluated based on their root mean squared error (RMSE) performance in estimating *R*_*w*_.

integrating water saturation profiles derived from multiple physics-based saturation models (based on water sample and core analyses) and an optimization-derived saturation curve obtained from equation 3 (second track from the right), as well as a differential curve comparing calculated versus measured deep resistivity (first track from the right).

**Fig. 10:**
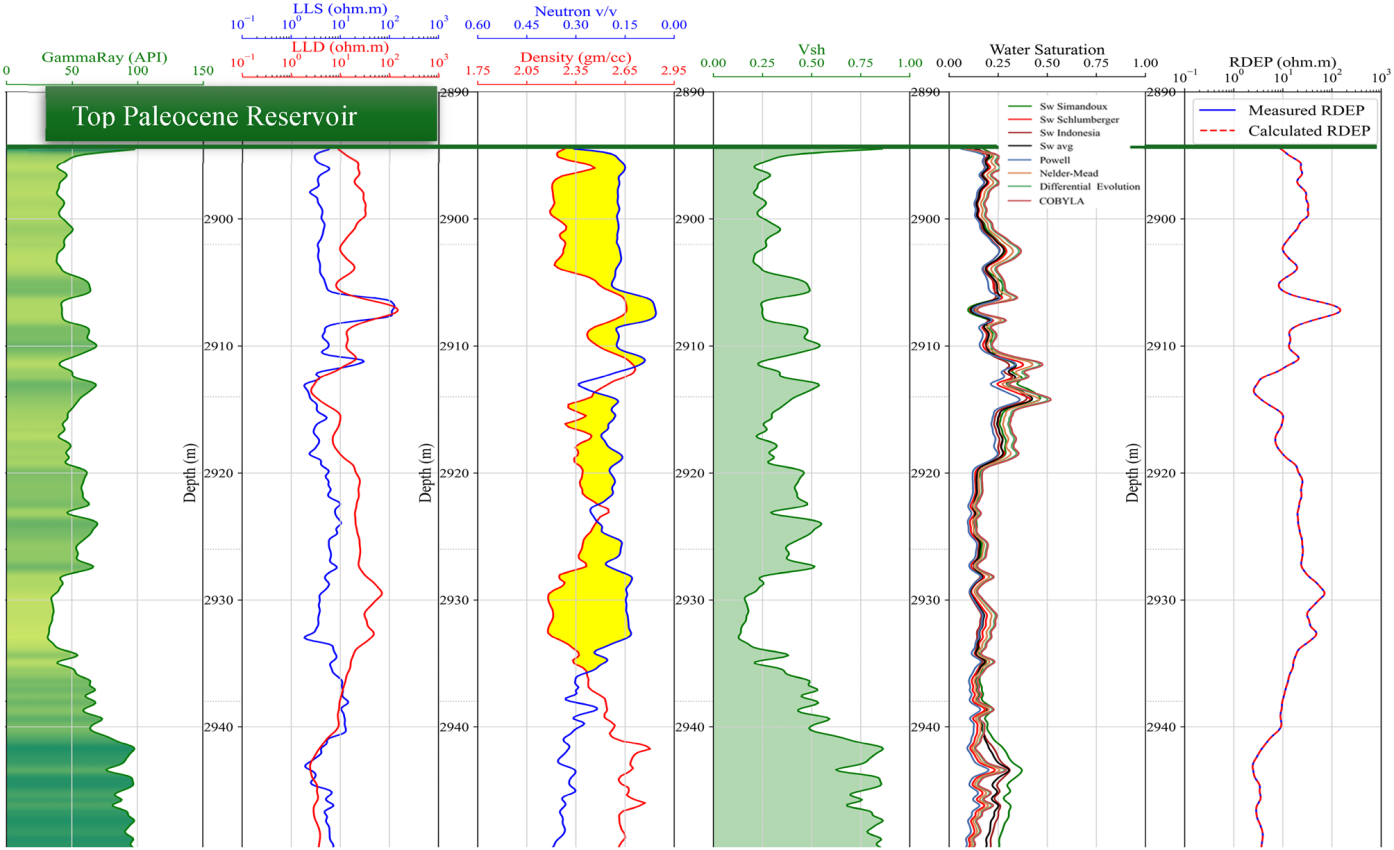
Composite Log Display for Well NS-02 Illustrating the Middle Jurassic Hugin and Sleipner Reservoirs.

Results comparison (Fig. 10) revealed that algorithms such as Powell and, in certain cases, Nelder-Mead consistently produced the lowest RMSE values. For instance, in well NS-01, Powell’s algorithm achieved an optimized Rw value of 0.04704, which is virtually indistinguishable from the true measured value of 0.047, as evidenced by an RMSE close to zero. By contrast, Differential Evolution, while robust in exploring the global search space and handling constraints respectively, exhibited slightly higher RMSE values in several wells. These trends underscore the importance of algorithm selection in balancing local refinement and global exploration are critical aspects in capturing complex reservoir heterogeneities and mitigating the influence of measurement noise.

**Fig. 11:**
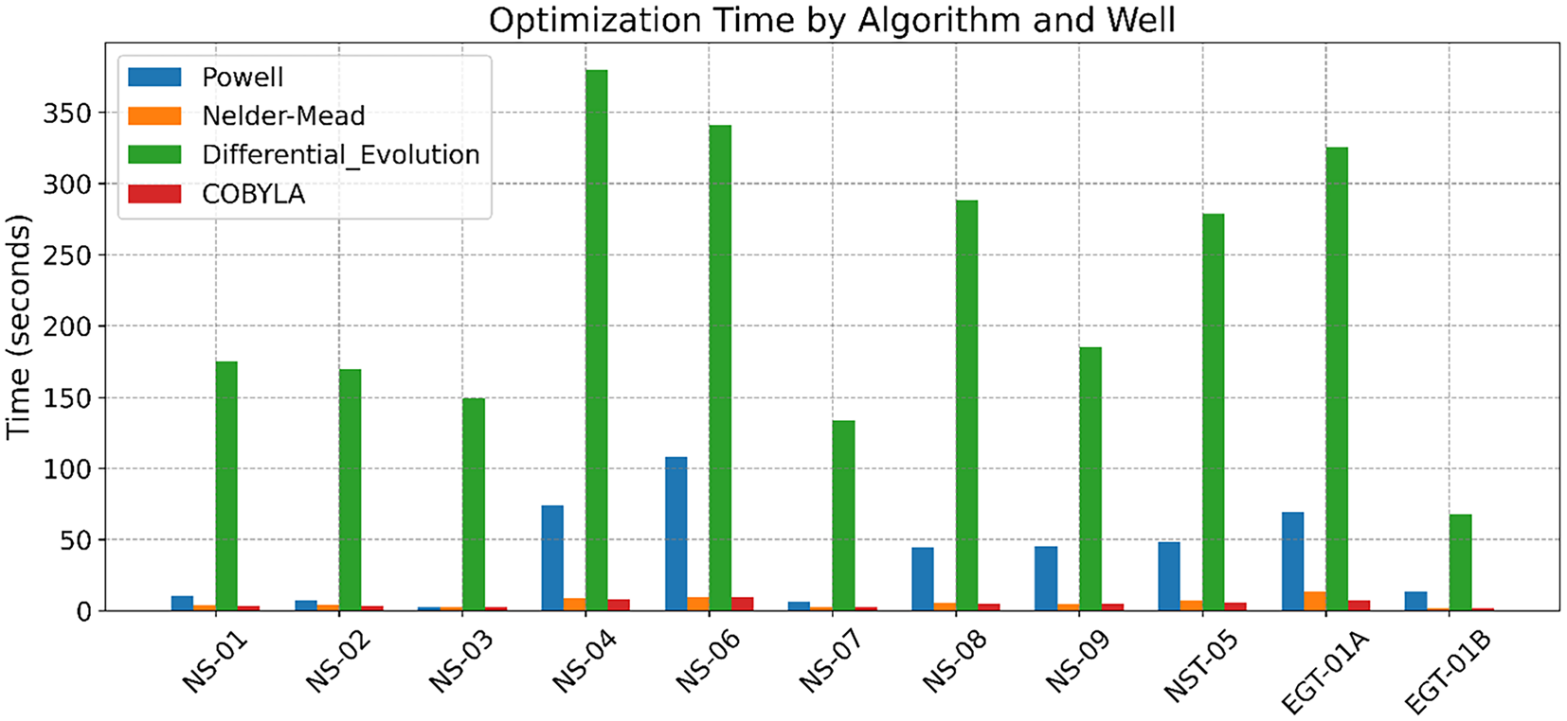
Computation time for each algorithm against all wells in the current study. Nelder-Mead with the lowest computational time algorithm and DE as the highest.

**Fig. 12:**
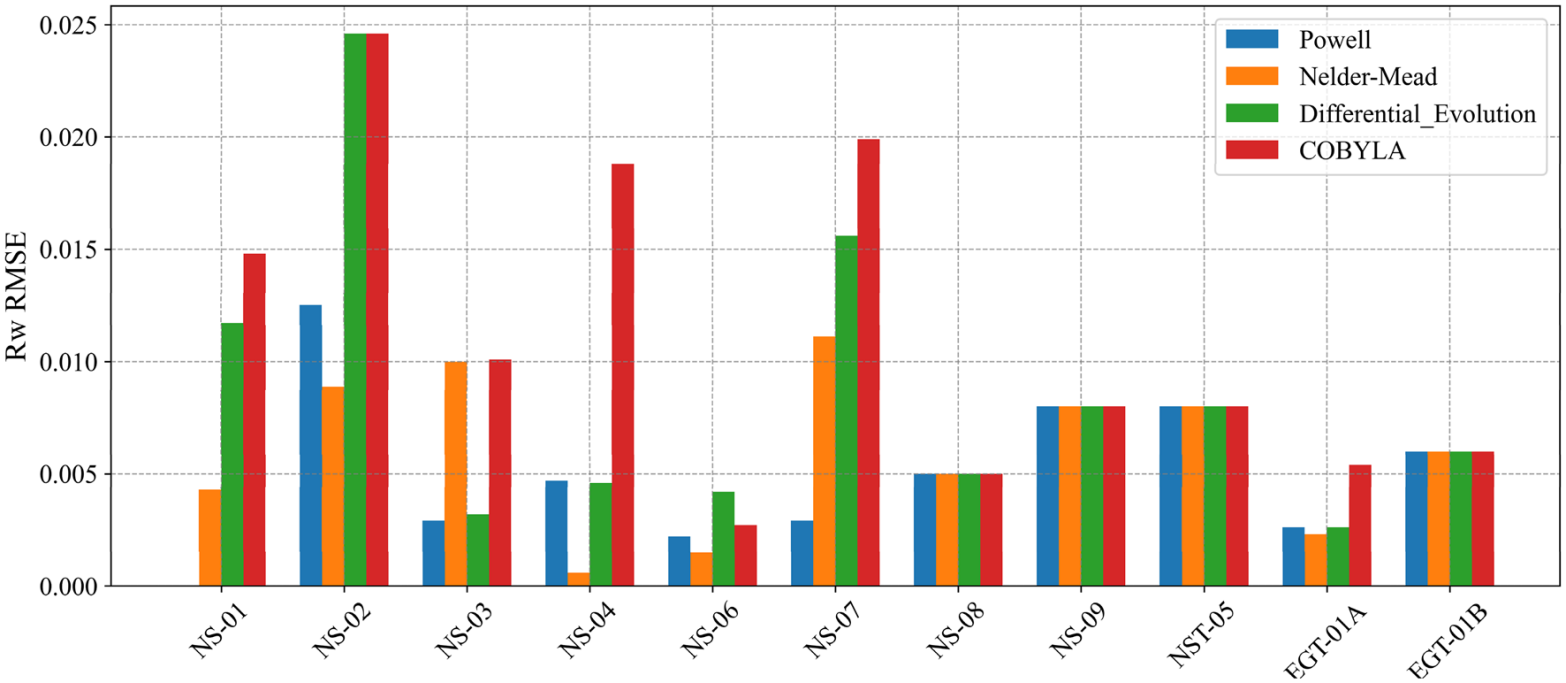
Root Mean Square Error (RMSE) of R_w_ for each well and algorithm quantifing the discrepancies between optimized *R*_*w*_ values and the true (measured) *R*_*w*_.

Table. 6 and 7 provide a detailed comparison that reinforces these observations. The numerical data reveal that the discrepancies between the optimized and true *R*_*w*_ values are minimized across most cases, with the Powell and Nelder-Mead algorithms frequently emerging as the most accurate estimator and recommended for such workflow based on both computation time and performance as highlighted in Figure. 11. Furthermore, Figure 12 graphically illustrates this trend by depicting error margins that are tightly clustered around low values, thereby visually confirming the efficacy of our optimization approach. These consistent results, achieved across diverse well conditions, suggest that our methodology is robust, effectively accommodating variations in local lithology and measurement inconsistencies. Minor deviations observed in well *NS-02* are likely attributable to residual noise within the well-log measurements, phenomena that our regularization and constraint-based framework is designed to mitigate but not entirely eliminate.

**Table 6:** Optimized formation water resistivity (*R*_*w*_) and associated RMSE for each well.

Well No.	True* Rw*	Optimized *Rw* (*Ω·m*)				RMSE (Optimized *Rw* vs True *Rw*)			
		Powell	Nelder-Mead	Differential Evolution	COBYLA	Powell	Nelder-Mead	Differential Evolution	COBYLA
NS-01	0.047	0.04704	0.05133	0.05870	0.06182	0.00001	0.0043	0.0117	0.0148
NS-02	0.045	0.03248	0.05391	0.06964	0.06964	0.0125	0.0089	0.0246	0.0246
NS-03	0.05	0.04708	0.0400	0.05315	0.03990	0.0029	0.0100	0.0032	0.0101
NS-04	0.051	0.05575	0.05160	0.06992	0.06980	0.0047	0.0006	0.0189	0.0188
NS-06	0.015	0.01717	0.01650	0.01919	0.01767	0.0022	0.0015	0.0042	0.0027
NS-07	0.05	0.04712	0.03894	0.06610	0.06990	0.0029	0.0111	0.0161	0.0199
NS-08	0.030	0.03500	0.03500	0.03501	0.35000	0.0050	0.0050	0.0050	0.0050
NS-09	0.045	0.02800	0.02800	0.02800	0.02800	0.0080	0.0080	0.0080	0.0080
NST-05	0.043	0.04000	0.04000	0.04000	0.04000	0.0030	0.0030	0.0030	0.0030
EGT-01A	0.027	0.02436	0.02475	0.02436	0.02162	0.0026	0.0023	0.0026	0.0054
EGT-02B	0.038	0.03100	0.03100	0.03100	0.03100	0.0060	0.0060	0.0060	0.0060

**Table 7:** RMSE between Optimized *S*_*w*_ and true *S*_*w*_.

Well No.	RMSE (Optimized *Sw* vs Core based Sw^*^)			
	Powell	Nelder-Mead	Differential Evolution	COBYLA
NS-01	0.0401	0.0346	0.0262	0.0231
NS-02	0.0669	0.0442	0.0478	0.0478
NS-03	0.0432	0.0668	0.0251	0.0671
NS-04	0.0368	0.0436	0.0334	0.0332
NS-06	0.0515	0.0566	0.0407	0.0482
NS-07	0.1110	0.1312	0.0732	0.0672
NS-08	0.1454	0.1454	0.1454	0.1454
NS-09	0.1693	0.1693	0.1693	0.1693
NST-05	0.1684	0.1684	0.1684	0.1684
EGT-01A	0.1287	0.1247	0.1287	0.1584
EGT-02B	0.1723	0.1723	0.1723	0.1723

^*^ *S*_*w*_ discussed in the table is the average *S*_*w*_ obtained from averaging *S*_*w*_ from three saturation models based on water samples and special core analysis to obtain (*R*_*w,*_* R*_*sh,*_* m, n, a*).

It is important to note that the near-zero RMSE values observed for the Powell algorithm (for *NS-01* well) represents a powerful validation of the methodology. This specific RMSE (Table 6) represents the absolute difference between the algorithm-derived *R*_*w*_ and the ground-truth *R*_*w*_ from core samples. This result demonstrates that the optimization framework is capable of accurately recovering the true formation water resistivity from the log data alone, serving as a robust physics-based anchor for the subsequent ML models.

**Fig. 13:**
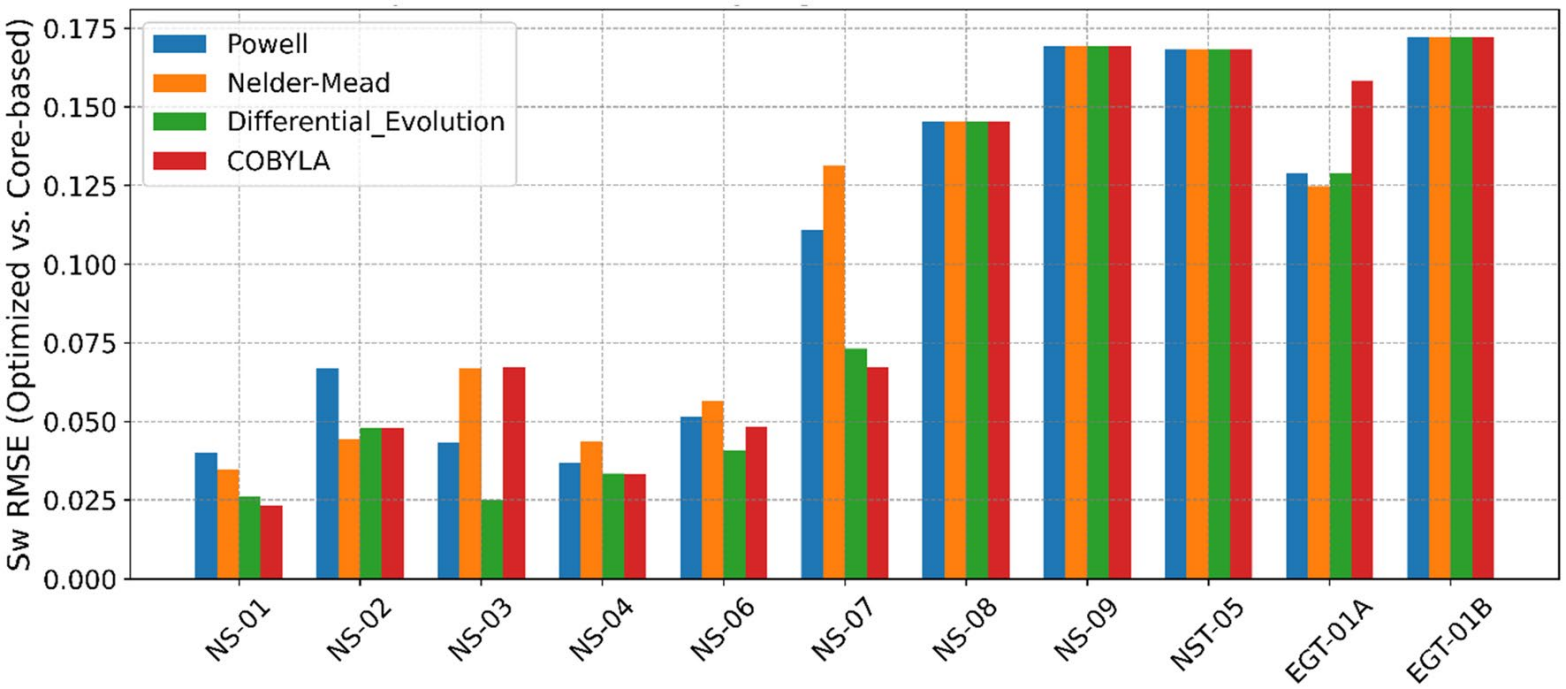
Root Mean Square Error (RMSE) of optimized Sw for each well and algorithm, quantifying the discrepancies between Sw derived from each optimization method and the Sw calculated from core-based parameters.

The validation of our optimized *S*_*w*_ values against conventional petrophysical models (Fig. 13) - obtained from core parameters - further substantiates the rigor of the optimization approach. When comparing our *S*_*w*_ estimates with those derived from established models such as Simandoux, Schlumberger, and Poupon-Leveaux, our results consistently exhibited low RMSE values (as detailed in Table 7). These results in error signifies not only a methodological improvement but also accurately capturing the underlying geophysical processes, particularly in accounting for shale-induced conductivity effects. These findings carry important geophysical implications, as even minor improvements in *S*_*w*_ estimation can substantially alter the interpretation of reservoir quality and hydrocarbon reserve calculations. The enhanced predictive accuracy afforded by our approach points to a significant advancement over conventional methods that require expensive and time-consuming coring and water samples.

### Machine learning results

**Fig. 14:**
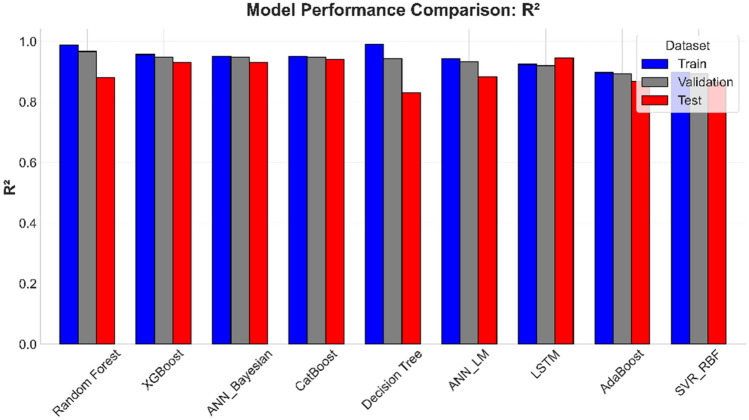
Comparison of the coefficient of determination across training, validation, and test Sets.

**Fig. 15:**
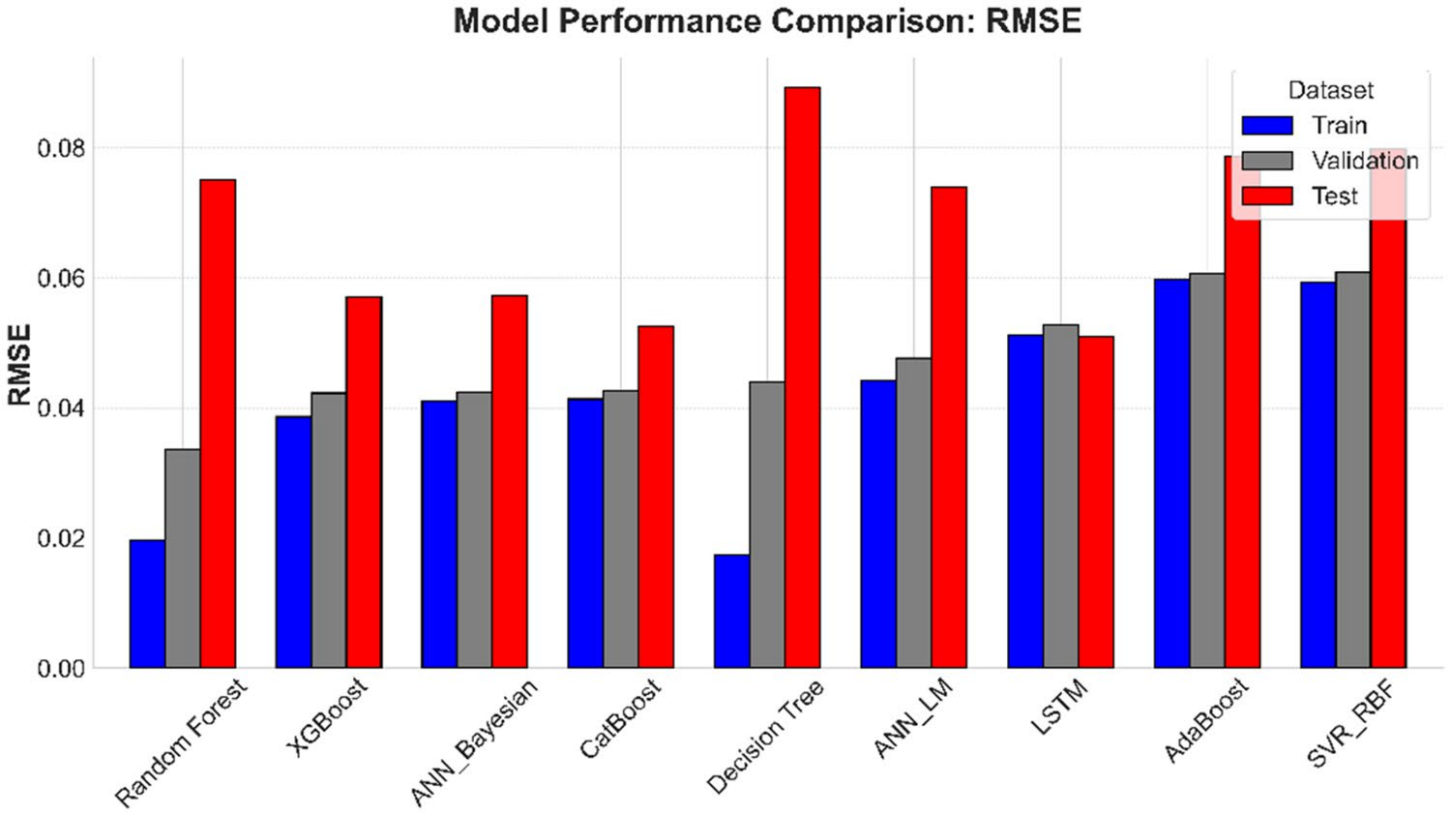
Comparison of root mean squared error across training, validation, and test sets.

**Fig. 16:**
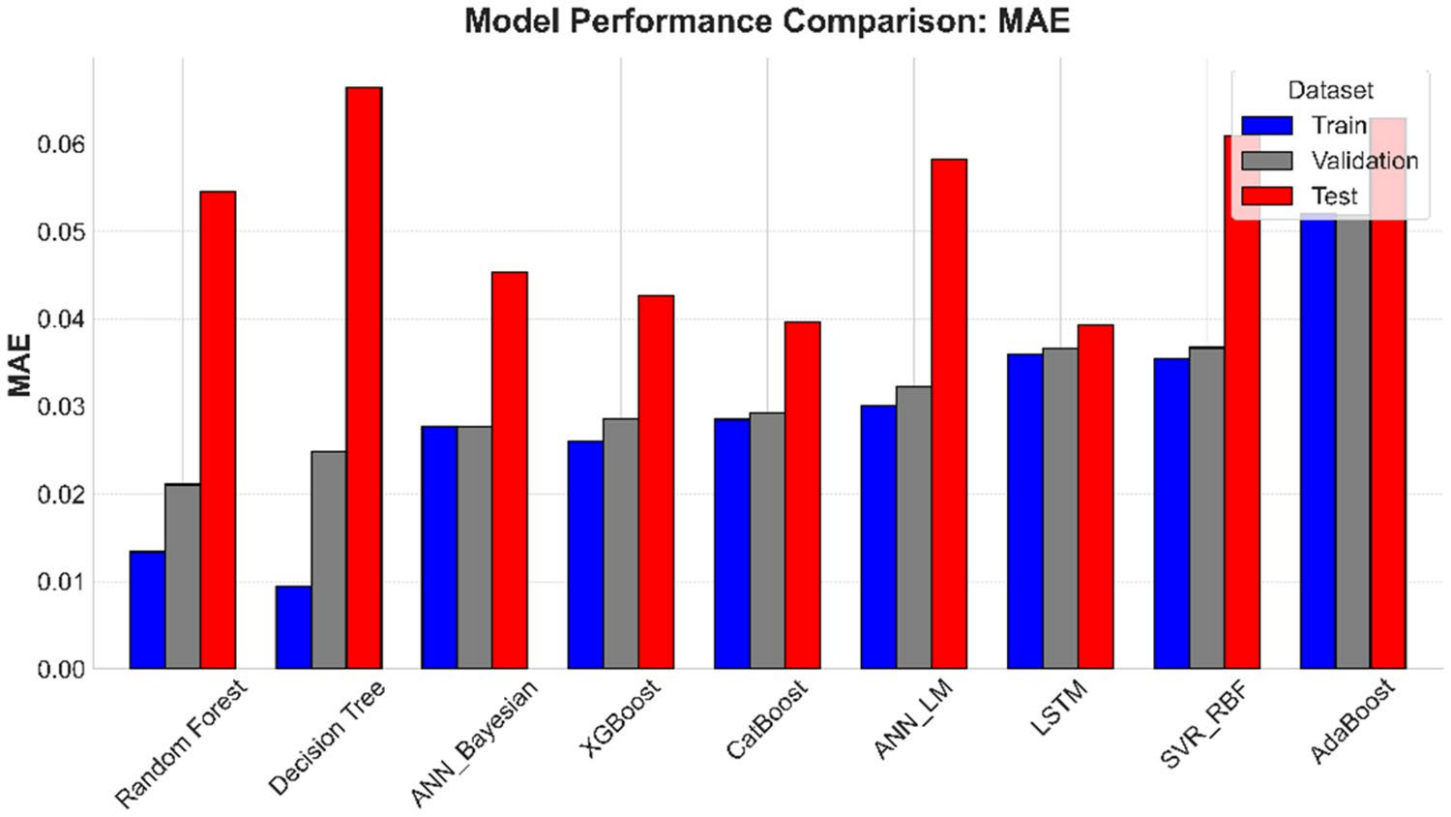
Comparison of Mean Absolute Error across training, validation, and test sets.

In parallel to the optimization framework, ML models developed for predicting *S*_*w*_ showcased robust performance across training, validation, and testing phases. A multi-metric evaluation (Figs. 14 - 16) and numerically summarized in Table 8 was undertaken using *MAE*, *RMSE*, and *R*^*2*^ to assess model fidelity. Tree-based ensemble methods - specifically Random Forest and XGBoost - demonstrated exceptional predictive accuracy, achieving CV-*R*^*2*^ values in the range of 0.967 and 0.9483 respectively, with low *MAE* and *RMSE* values. These results indicate that ensemble methods, benefiting from techniques such as bagging and subspace sampling, are particularly effective in reducing overfitting and capturing non-linear interdependencies within the data. Neural network approaches, including both feedforward architectures optimized via Bayesian as well as Levenberg-Marquardt designed for sequential data analysis, also exhibited strong performance with *R*^*2*^ scores consistently in range of 0.9481 to 0.9331. The slight differences in performance between ensemble methods and neural network models are reflective of inherent methodological strengths; whereas ensemble models benefit from reduced variance via aggregation, the neural networks are adept at modeling complex, depth-dependent patterns in the logs.

**Table 8:**
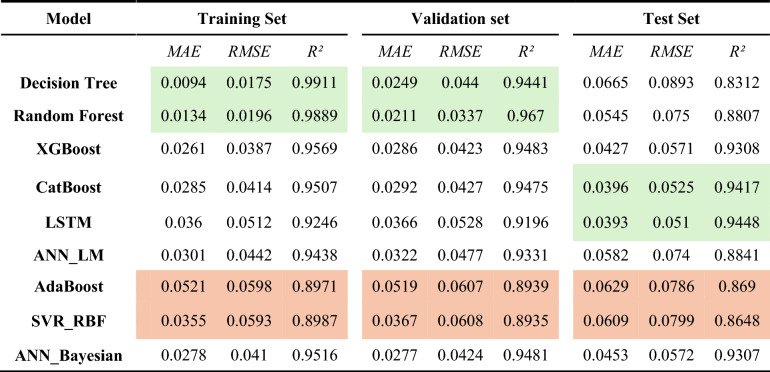
Performance metrics of ML Models across training, validation, and test Sets.

Green filled cells represent best two candidates best on performance metrics. While red cells representing lowest two models in performance.

Fig. 16.Comparison of Mean Absolute Error across training, validation, and test sets.Fig. 16.Comparison of Mean Absolute Error across training, validation, and test sets.Fig. 16.Comparison of Mean Absolute Error across training, validation, and test sets.

**Fig. 17:**
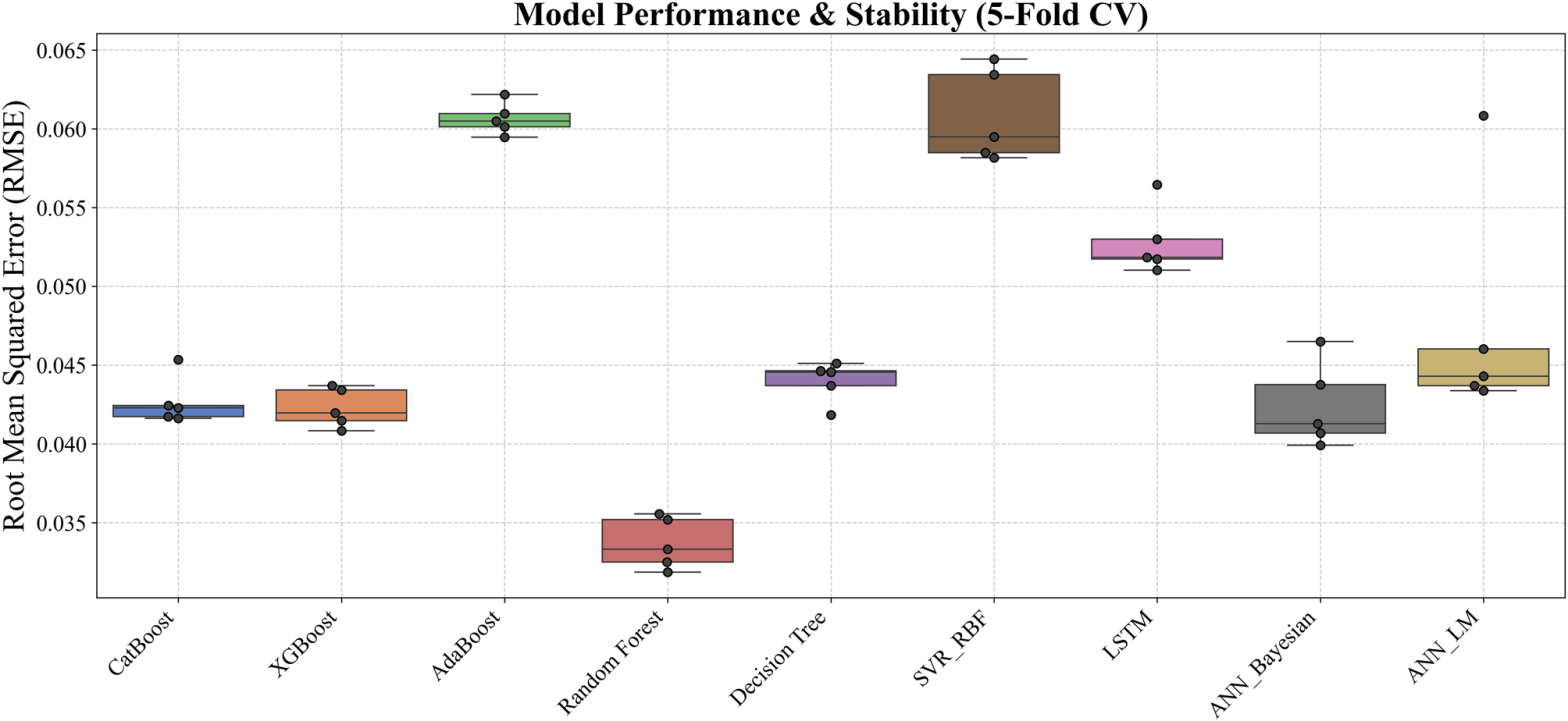
Boxplot comparison of 5-fold cross-validation RMSE for all ML models. The box shows the interquartile range (IQR), the line indicates the median, and the whiskers represent the full range of the 5-fold scores, providing a clear view of model performance and stability.

The stability and generalizability of these ML models were further investigated by a rigorous 5-fold cross-validation protocol, and its results are visualized in Figure 17. This methodological rigor ensured that performance metrics obtained on the training data closely mirrored those seen in the validation sets, thereby providing confidence that the models are well-calibrated for unseen data. When subjected to independent test sets - comprising wells from both the Norwegian North Sea and the Egyptian Western Desert - the models retained high predictive power, confirming that the integration of robust cross-validation techniques and stringent hyperparameter tuning (as detailed in Table 4) has resulted in models with reliable generalizability across varied geological contexts. Graphical illustrations spanning Figures 7 through 9 vividly capture these trends by juxtaposing actual versus predicted *S*_*w*_ values, with error analyses revealing minimal deviations even in instances where complex lithological transitions may challenge predictive accuracy.

**Fig. 18:**
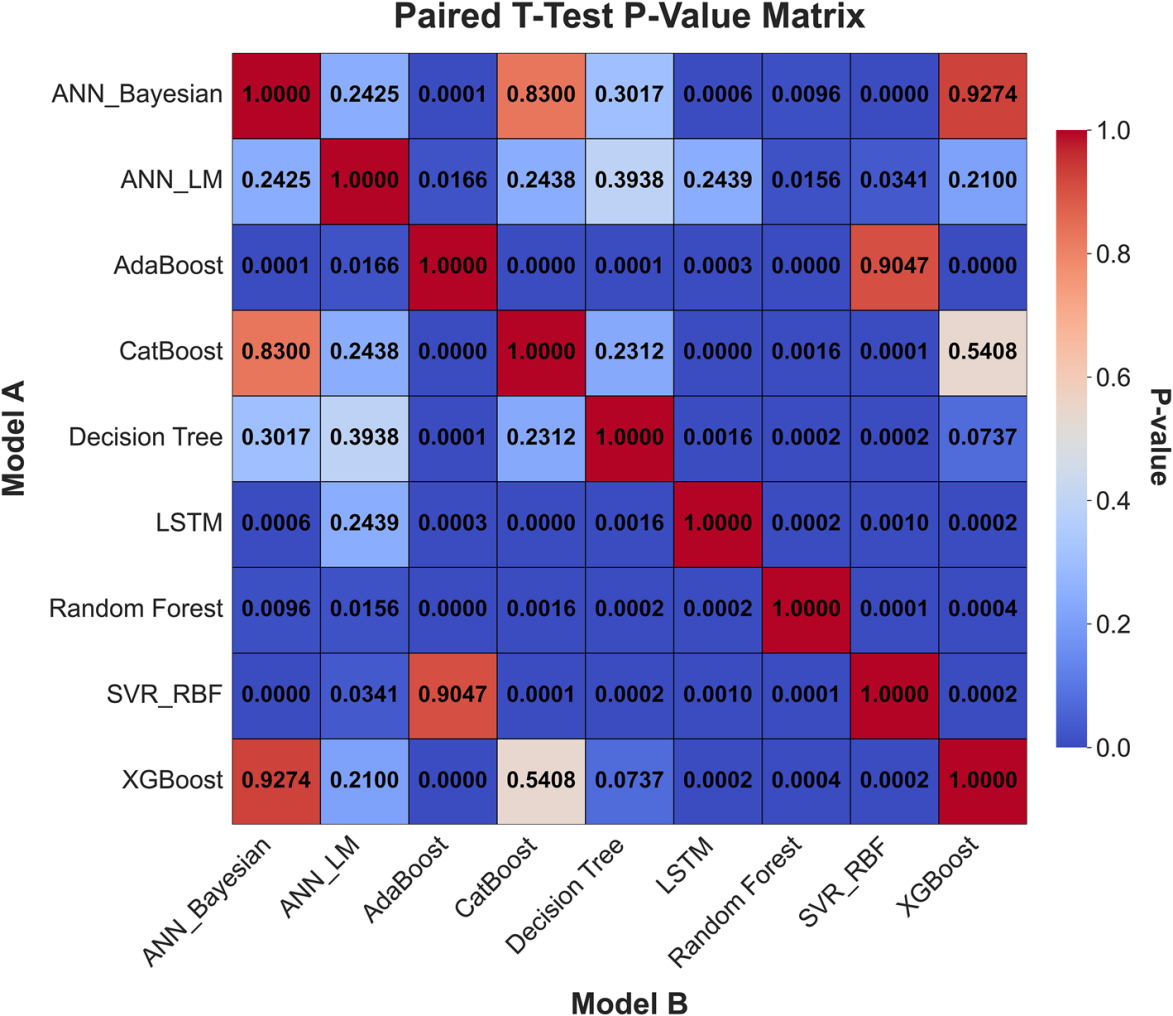
Boxplot comparison of 5-fold cross-validation RMSE for all ML models. The box shows the interquartile range (IQR), the line indicates the median, and the whiskers represent the full range of the 5-fold scores, providing a clear view of model performance and stability.

Comprehensive statistical validation of the nine developed models was conducted using a 5-fold cross-validation framework, with model performance differentials assessed via paired t-tests (at $\alpha =0.05$) (Fig. 18). This rigorous analysis, based on the mean Root Mean Squared Error (*RMSE*) of water saturation, established a definitive performance hierarchy. The Random Forest (*RF*) model (*RMSE = 0.0337*) emerged as the statistically dominant architecture, demonstrating a significantly lower prediction error than all other evaluated algorithms ($p \le 0.0017$ in all comparisons). A distinct, high-performance cluster was also identified, comprising XGBoost (*RMSE = 0.0423*), CatBoost (*RMSE = 0.0427*), ANN_Bayesian (*RMSE = 0.0424*), and Decision Tree (*RMSE = 0.044*). These models, while statistically outperformed by RF, exhibited no significant performance difference relative to one another (e.g., CatBoost vs. XGBoost, *p = 0.540*; XGBoost vs. Decision Tree, *p = 0.073*), confirming their equivalent and robust predictive capabilities for this application.

Conversely, the analysis statistically segregated the remaining models into lower-performance tiers. The ANN_LM (*RMSE = 0.0477*) and LSTM (*RMSE = 0.0528*) models formed an intermediate group, performing statistically similarly to each other (*p = 0.243*) but proving significantly less accurate than the top-tier ensemble and Bayesian architectures. At the lowest performance bound, the AdaBoost (RMSE = 0.0607) and SVR_RBF (*RMSE = 0.0608*) models were found to be statistically indistinguishable (*p = 0.904*). These two models, however, demonstrated a significantly inferior predictive accuracy compared to all seven other models in the study, confirming their in-suitability for this specific subsurface characterization problem. This statistical validation provides a clear mandate for the selection of Random Forest, or models from the equivalent XGBoost/CatBoost cluster, for deployment.

This statistical analysis creates a clear narrative when compared with the final blind test set performance (Table 8). The Random Forest model, which was the statistically undisputed winner of the cross-validation, showed a significant drop in performance on the unseen test data (Test R^2^: 0.880). This indicates that it overfit to the Norwegian dataset. Conversely, the models in the "second-tier" of CV performance - LSTM (Test R^2^: 0.9448), CatBoost (Test R^2^: 0.9417), ANN_Bayesian (Test R^2^: 0.9307), and XGBoost (Test R^2^: 0.9308) - all demonstrated good and stable generalization, outperforming the overfit Random Forest model significantly. This result is crucial, as it confirms that the gradient-boosted ensemble methods and well-tuned neural networks are the most robust and reliable for this framework, while the standard Random Forest, despite its high CV score, is less generalizable.

A salient aspect of our integrated methodology is the model interpretability analysis, for which we employed SHAP (SHapley Additive exPlanations). This advanced technique provides a robust, model-specific measure of feature importance, superseding the simple linear correlation of a relevancy factor. These SHAP summary plots (Fig. 5) confirm that *RDEP,NPHI,and* V_*sh*__GR are consistently the most impactful features, as expected from petrophysical principles. However, SHAP also reveals complex non-linear interactions. For example, high NPHI (high porosity) and low RHOB (low density) both correctly contribute positively to higher predicted Sw (as they indicate water-filled pore space), but their relative importance varies between different model architectures. This analysis confirms that all four input features are critical and that the models successfully learned the underlying, non-linear petrophysical relationships.

**Fig. 19:**
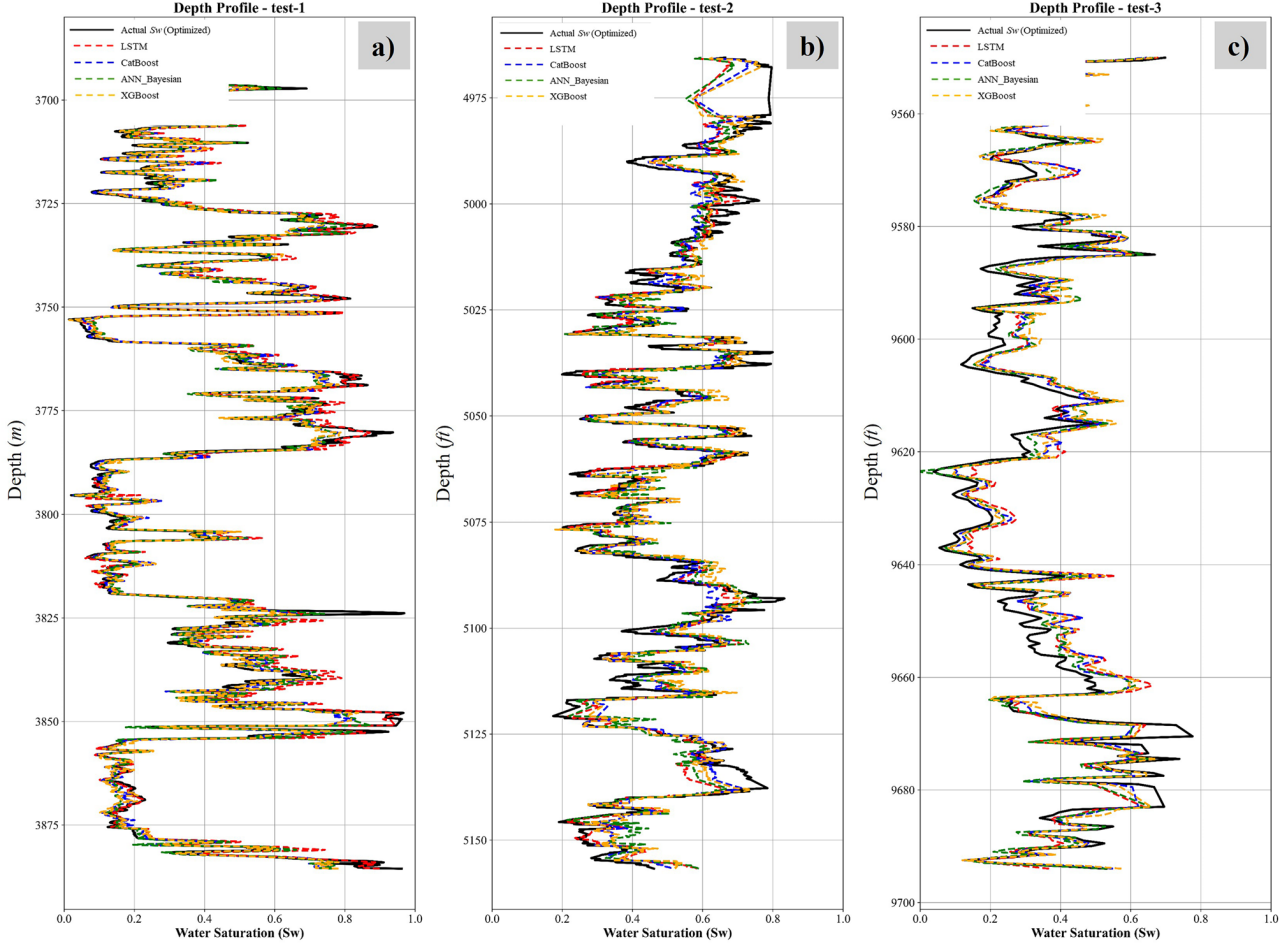
Testing results comparing optimization-derived versus Machine Learning-predicted *S*_*w*_. (**a**) well NST-05 from Norway north sea (**b**) well EGT-01A (Egypt) Matruh - Shushan Basin (**c**) well EGT-02B (Egypt) Abu El-Gharadig basin.

The results from the three independent test wells (Fig. 19) conclusively validate the generalizability and robustness of the proposed integrated framework, demonstrating its capacity to resolve spatial heterogeneity in *S*_*w*_ across distinct geological provinces. For well *NST-05* (Norwegian North Sea), the ML models achieved exceptional predictive accuracy (RMSE = 0.04, MAE = 0.030, and R^2^ = 0.97) for LSTM algorithm, attributable to the synergistic alignment between the training dataset lithostratigraphic features (E. Jurassic Statfjord Formation) and the test well depositional environment. This consistency in geological setting enabled the algorithms to effectively map the nonlinear relationships between input petrophysical variables and optimization-derived *S*_*w*_, leveraging learned patterns from analogous facies architectures.

In contrast, Wells *EGT-01A* (Egyptian Matruh-Shushan Basin) and *EGT-02B* (Abu El-Gharadig Basin) presented a more rigorous validation scenario, as these reservoirs - characterized by upper cretaceous Bahariya and Jurassic Safa formations - were entirely excluded from the training corpus. Despite this deliberate exclusion, the models exhibited commendable generalizability. The marginal reduction in accuracy relative to Well NST-05 reflects inherent domain adaptation challenges, including covariate possible shifts in shale mineralogy and disparities in diagenetic overprints. Crucially, this performance was achieved without reliance on core-calibrated parameters or formation water samples, underscoring the ability of our framework to decode latent petrophysical interactions solely from conventional log suites.

The differential in predictive fidelity across basins highlights a critical trade-off: while training data homogeneity enhances model precision, the proposed methodology retains sufficient flexibility to extrapolate beyond its immediate experiential domain. This balance positions the framework as a pragmatic tool for frontier exploration, where core data scarcity and lithological novelty often constrain conventional interpretation workflows. The results collectively affirm that the integration of optimization-derived *S*_*w*_ targets with ensemble ML architectures mitigates overfitting risks while preserving sensitivity to subtle, depth-dependent reservoir signatures a paradigm shift toward scalable, data-driven petrophysics.

The broader implications of integrating optimization and machine learning frameworks are profound. By delivering more reliable water saturation estimates, our approach provides reservoir engineers with a powerful tool for accurate hydrocarbon reserve evaluation and reservoir performance forecasting. The operational advantages are clear: reduced dependency on extensive core analyses, enhanced cost-effectiveness, and a marked improvement in the generalizability of petrophysical evaluations across diverse formations. In direct comparison with conventional empirical and graphical methods - such as Archie-based interpretations - the proposed framework overcomes intrinsic limitations by offering a dynamic, data-centric approach that captures the inherent complexities of heterogeneous reservoirs.

### Strengths of the integrated framework

A primary strength of this framework is its integrated, data-driven, two-stage "optimization-first" approach. By first optimizing for a single, global *R*_*w*_, we anchor the subsequent *S*_*w*_ prediction in a physically constrained parameter. This optimized *S*_*w*_ serves as a high-quality, continuous "pseudo-core" label for the ML models, removing the reliance on sparse and expensive core data. A second strength is the rigorous validation across geologically distinct basins (Norwegian North Sea and Egyptian Western Desert), which intentionally mimics a realistic “scarce data” scenario and demonstrates the framework ability to generalize beyond its training domain.

### Limitations and future work

Nevertheless, despite the notable successes, several limitations warrant acknowledgement and provide direction for future research.

- Dataset Size and Generalizability: The training dataset, while representative, is limited to 8 wells from one basin. Despite successfully mimics a scarce-data exploration scenario. However, scaling this framework for other, more complex geological settings (e.g., carbonates, volcaniclastics) would require further testing. A promising avenue for future work is the application of transfer learning, where a model could be pre-trained on a large public dataset and then fine-tuned using only one or two wells from a new frontier basin.
- Optimization Sensitivity: The optimization results (Very low RMSE) is a key strength, but it may also be sensitive to poor data quality. Future work should include a sensitivity analysis to test the optimization robustness to varying levels of data noise, which could cause convergence to a non-representative local minimum.
- CV Reproducibility: As discussed our 5-fold cross-validation protocol used a single fixed random seed for reproducibility. Future work could involve running the CV multiple times with different random seeds to provide a statistical distribution of model performance and a more robust measure of variance.
- Overfitting: Minor instances of overfitting, particularly the significant case of the Random Forest model, suggest that continued refinement in hyperparameter tuning and model architecture is necessary.

## Conclusion

This study successfully developed and validated an innovative, two-stage integrated framework that couples numerical optimization with machine learning to accurately characterize shaley-sand reservoirs. Our primary contribution is the 'optimization-first’ workflow: by first optimizing for a single, physically-constrained global Rw, we generate a continuous, high-fidelity 'pseudo-core’ Sw log. This approach was validated by the Powell algorithm, which consistently recovered the true Rw from log data with near-zero error (e.g., 1×10^−4^ RMSE) against measured samples, confirming its robustness.

This high-quality optimized *S*_*w*_ log served as the training target for a comprehensive suite of ML models. The results revealed a critical insight into model selection, validated by statistical (paired t-test) and blind-well analyses. While Random Forest model was the statistically significant winner during cross-validation (CV R^2^: 0.967), it failed to generalize to the blind test data (Test R^2^: 0.88), indicating it had overfit to the training dataset. Conversely, the gradient-boosting ensembles, specifically CatBoost (Test R^2^: 0.942), XGBoost (Test R^2^: 0.9308) and well-tuned neural networks like LSTM (Test R^2^: 0.945), and ANN_ Bayesian (Test R^2^: 0.9307) demonstrated superior and more stable generalization. This finding underscores that "Optimization + LSTM/ANN_Bayesian " and "Optimization + CatBoost/XGBoost" hybrid as the most reliable and recommended workflow.

Our interpretability analysis using SHAP confirmed that all models successfully learned the complex, non-linear petrophysical relationships, identifying and *RDEP, NPHI, and* V_sh_*_GR* as the most impactful features, as expected. Beyond technical accomplishments, this framework offers tangible operational advantages. By demonstrating that a robust model can be trained in a “scarce data” scenario and successfully deployed in a completely different geological basin, it provides a cost-effective and scalable alternative to traditional workflows. This methodology significantly reduces reliance on expensive core analyses, fostering improved accuracy in hydrocarbon reserve evaluations.

While this research marks a significant step toward more data-centric petrophysics, we acknowledge the path for future work. This includes exploring transfer learning to fine-tune models for new basins and conducting further sensitivity analysis on the optimization performance in noisy log environments with utilization of metaheuristics optimization algorithms paving the way for wider adoption of robust, data-driven workflows in reservoir characterization.

## Supplementary Information

Supplementary Information.

## Data Availability

Norwegian dataset analyzed during this study are included in this published article [and its supplementary information files].The testing datasets from the western desert and python codes used in the current study available from the corresponding author on reasonable request.
