# Supplementary material for: Integrating optimization and machine learning for estimating water resistivity and saturation in shaley sand reservoirs
Source: Sci Rep. 2026 Feb 11;16:6342. doi: 10.1038/s41598-026-36133-w (PMC12905170; doi:10.1038/s41598-026-36133-w)
Supplement: Supplementary file 1 — Supplementary Information. [file 41598_2026_36133_MOESM1_ESM.pdf]

SPRINGER NATURE

Change of authorship request form - Journals (pre-acceptance)

Important information. Please read.

- This form should be used by authors to request any change in authorship (adding/deleting authors) including changes in corresponding authors. This form should not be used for name changes. Please fully complete all sections. Use black ink and block capitals and provide each author's full name with the given name first followed by the family name.

- By signing this declaration, all authors guarantee that the order of the authors are in accordance with their scientific contribution, if applicable as different conventions apply per discipline, and that only authors have been added who made a meaningful contribution to the work.

- Please note, in author collaborations where there is formal agreement for representing the collaboration, it is sufficient for the representative or legal guarantor (usually the corresponding author) to complete and sign the Authorship Change Form on behalf of all authors, **next to the added/removed author(s)**. **(Complete Section 3, followed by Section 6.)**
  In author collaborations where there is no formal agreement for representing the collaboration and **there are more than 10 authors**, one may sign for all, provided the signer appends correspondence that attests that each of the authors have agreed to the change **and the added/removed authors sign the form**. **(Complete Section 3, followed by Section 6.)**

- Please note, we cannot investigate or mediate any authorship disputes. If you are unable to obtain agreement from all authors (including those who you wish to be removed) you must refer the matter to your institution(s) for investigation. Please inform us if you need to do this.

- If you are not able to return a fully completed form within **30 days** of the date that it was sent to the author requesting the change, we may have to withdraw your manuscript. We cannot publish manuscripts where authorship has not been agreed by all authors (including those who have been removed).

- Incomplete forms will be rejected.

- Please return/upload this form, fully completed, to the Journals Editorial Office. The Journal and/or Publisher will consider the information you have provided to decide whether to approve the proposed change in authorship. We may decide to contact your institution for more information or undertake a further investigation, if appropriate, before making a final decision.

SPRINGER NATURE Springer Nature is one of the world's leading global research, educational and professional publishers, created in May 2015 through the combination of Nature Publishing Group, Palgrave Macmillan, Macmillan Education and Springer Science+Business Media.

SPRINGER NATURE

Change of authorship request form - Journals (pre-acceptance)

Section 1: Please provide the current title of manuscript

Manuscript ID no.: d4394304-47d2-4360-9092-a4a16ba7937f v2.0

Title: Integrating Optimization and Machine Learning for Estimating Water Resistivity and Saturation in Shaley Sand Reservoirs

Section 2: Please provide the previous authorship, in the order shown on the manuscript before the changes were introduced. Please indicate the corresponding author by adding (CA) behind the name.

	First name(s)	Family name	ORCID or SCOPUS id, if available
1st author	Muhammad	El Hameedy (CA)	0000-0002-8987-3224
2nd author	Ahmed	El-Khteeb	0009-0001-1215-7027
3rd author	Walid	Mabrouk	0000-0001-7048-314
4th author	Ahmed	Metwally	0000-0002-6079-294
5th author			
6th author			
7th author			
8th author			
9th author			
10th author			

Please use an additional sheet if there are more than 10 authors.

SPRINGER NATURE Springer Nature is one of the world's leading global research, educational and professional publishers, created in May 2015 through the combination of Nature Publishing Group, Palgrave Macmillan, Macmillan Education and Springer Science+Business Media.

SPRINGER NATURE

Change of authorship request form - Journals (pre-acceptance)

Section 3: Please provide a justification for change. Please use this section to explain your reasons for changing the authorship of your manuscript, e.g. what necessitated the change in authorship? Please refer to the (journal) policy pages for more information about authorship. Please explain why omitted authors were not originally included and/or why authors were removed on the submitted manuscript.

The authorship of this manuscript has been modified by removing Dr. Ahmed El-Khteeb to comply with a new, recently enacted institutional policy at our department (Department of Geophysics, Faculty of Science, Cairo University).

This manuscript is a primary component of the doctoral thesis for the corresponding author, Muhammad A. El Hameedy. Dr. Ahmed El-Khteeb is also a registered PhD candidate within the same department. A new departmental regulation now stipulates that for a research paper to be permissible as a core chapter of a PhD candidate's thesis, it may not list other active PhD candidates as co-authors.

To ensure this manuscript complies with this new institutional requirement for graduation, we have had to remove Dr. El-Khteeb from the author byline.

We wish to confirm that this change is purely administrative and not the result of a scientific or authorship dispute. Dr. El-Khteeb was involved in the initial stages of the research, and we are grateful for his contribution. He has been fully informed of this situation and the policy requirement, and he has provided his full consent and agreement for his removal from the author list. His contribution will be appropriately recognized in the manuscript acknowledgments section.

Section 4: Proposed new authorship. Please provide your new authorship list in the order you would like it to appear on the manuscript. Please indicate the corresponding author by adding (CA) behind the name. <u>If the Corresponding Author has changed, please indicate the reason under section 3</u>.

	First name(s)	Family name (this name will appear in full on the final publication and will be searchable in various abstract and indexing databases)	Affiliated institute	E-mail address
1st author	Muhammad	El Hameedy (CA)	Cairo University	mashraf@sci.cu.edu.eg
2nd author	Walid	Mabrouk	Cairo University	wmabrouk@sci.cu.edu.eg
3rd author	Ahmed	Metwally	Cairo University	amohesn@sci.cu.edu.eg
4th author				
5th author				
6th author				
7th author				
8th author				
9th author				
10th author				

Please use an additional sheet if there are more than 10 authors.

SPRINGER NATURE Springer Nature is one of the world's leading global research, educational and professional publishers, created in May 2015 through the combination of Nature Publishing Group, Palgrave Macmillan, Macmillan Education and Springer Science+Business Media.

Change of authorship request form - Journals (pre-acceptance)

Section 5: Author contribution, Acknowledgement and Disclosures. Please use this section to provide a new disclosure statement and, if appropriate, acknowledge any contributors who have been removed as authors and ensure you state what contribution any new authors made (if applicable per the journal or book (series) policy). Please ensure these are updated in your manuscript - after approval of the change(s) - as our production department will not transfer the information in this form to your manuscript.

New acknowledgements:

The authors wish to express their gratitude to Dr. Ahmed El-Khteeb for his valuable discussions and contributions during the initial stages of this research. We are also deeply grateful to the anonymous reviewers for their insightful and constructive comments. Their feedback significantly improved the clarity, rigor, and overall quality of this manuscript.

New Disclosures (financial and non-financial interests, funding):

FUNDING:
The authors declare that no funds, grants, or other support were received during the preparation of this manuscript.
CONFLICT OF INTEREST:
The authors have no relevant financial or non-financial interests to disclose.

New Author Contributions statement (if applicable per the journal policy):

M.A.E.: Led the conceptualization, designed the complete methodology, and developed the Python software for the optimization and machine learning pipelines, performed t analysis, data curation, and validation, and the primary author, writing the original draft, creating all visualizations. W.M.M.: Contributed to the study's initial conceptualization formal supervision, and critically reviewed and edited the manuscript. A.M.M.: Contributed to the study's conceptualization, provided overall project administration and super critically reviewed and edited the final manuscript.

State 'Not applicable' if there are no new authors.

SPRINGER NATURE Springer Nature is one of the world's leading global research, educational and professional publishers, created in May 2015 through the combination of Nature Publishing Group, Palgrave Macmillan, Macmillan Education and Springer Science+Business Media.

SPRINGER NATURE

Change of authorship request form - Journals (pre-acceptance)

Section 6: Declaration of agreement. *All* authors, unchanged, new and removed *must* sign this declaration.
(NB: Please print the form, (docu)-sign and return/upload a scanned copy. Please note that signatures that have been inserted as an image file are acceptable as long as it is handwritten. Typed names in the signature box are unacceptable.) *** Please delete as appropriate. Delete all of the bold if you were on the original authorship list and are remaining as an author.**

	First name	Family name		Signature	Date
1st author	Muhammad	El Hameedy (CA)	I agree to the proposed new authorshin shown in section 4		13/11/2025
2nd author	Ahmed	El-Khteeb	I agree to the proposed new authorship shown in section 4 **removal*of my name to the authorship list**		13/11/2025
3rd author	Walid	Mabrouk	I agree to the proposed new authorship shown in section 4		13/11/2025
4th authors	Ahmed	Metwally	I agree to the proposed new authorship shown in section 4		13/11/2025
5th author			I agree to the proposed new authorship shown in section 4 **/and the addition/removal*of my name to the authorship list** /and the proposed change in corresponding author		
6th author			I agree to the proposed new authorship shown in section 4 **/and the addition/removal*of my name to the authorship list** /and the proposed change in corresponding author		
7th author			I agree to the proposed new authorship shown in section 4 **/and the addition/removal*of my name to the authorship list** /and the proposed change in corresponding author		

SPRINGER NATURE Springer Nature is one of the world's leading global research, educational and professional publishers, created in May 2015 through the combination of Nature Publishing Group, Palgrave Macmillan, Macmillan Education and Springer Science+Business Media.

	First name	Family name		Signature	Date
8th author			I agree to the proposed new authorship shown in section 4 /**and the addition/removal*of my name to the authorship list** /and the proposed change in corresponding author		
9th author			I agree to the proposed new authorship shown in section 4 /**and the addition/removal*of my name to the authorship list** /and the proposed change in corresponding author		
10th author			I agree to the proposed new authorship shown in section 4 /**and the addition/removal*of my name to the authorship list** /and the proposed change in corresponding author		

Please use an additional sheet if there are more than 10 authors.

In case of author collaborations with formal agreement:

	Name of consortium/consortia	First name	Family name		Signature	Date
Representative/legal guarantor				I agree to the proposed new authorship shown in section 4 /**and the addition/removal*of my name to the authorship list** /and the proposed change in corresponding author		

Both added/removed authors should complete the information in the first table under Section 6.

---- End of form ----

SPRINGER NATURE Springer Nature is one of the world's leading global research, educational and professional publishers, created in May 2015 through the combination of Nature Publishing Group, Palgrave Macmillan, Macmillan Education and Springer Science+Business Media.
